# Artemether ameliorates kidney injury by restoring redox imbalance and improving mitochondrial function in Adriamycin nephropathy in mice

**DOI:** 10.1038/s41598-020-80298-x

**Published:** 2021-01-14

**Authors:** Pengxun Han, Yuchun Cai, Yao Wang, Wenci Weng, Yinghui Chen, Menghua Wang, Hongyue Zhan, Xuewen Yu, Taifen Wang, Mumin Shao, Huili Sun

**Affiliations:** 1 Department of Nephrology, Shenzhen Traditional Chinese Medicine Hospital, The Fourth Clinical Medical College of Guangzhou University of Chinese Medicine, 1 Fuhua Road, Futian District, Shenzhen, 518033 Guangdong China; 2 Department of Pathology, Shenzhen Traditional Chinese Medicine Hospital, The Fourth Clinical Medical College of Guangzhou University of Chinese Medicine, 1 Fuhua Road, Futian District, Shenzhen, 518033 Guangdong China

**Keywords:** Medical research, Nephrology

## Abstract

The kidney is a high-energy demand organ rich in mitochondria especially renal tubular cells. Emerging evidence suggests that mitochondrial dysfunction, redox imbalance and kidney injury are interconnected. Artemether has biological effects by targeting mitochondria and exhibits potential therapeutic value for kidney disease. However, the underlying molecular mechanisms have not been fully elucidated. This study was performed to determine the effects of artemether on Adriamycin-induced nephropathy and the potential mechanisms were also investigated. In vivo, an Adriamycin nephropathy mouse model was established, and mice were treated with or without artemether for 2 weeks. In vitro, NRK-52E cells were stimulated with TGF-β1 and treated with or without artemether for 24 h. Then renal damage and cell changes were evaluated. The results demonstrated that artemether reduced urinary protein excretion, recovered podocyte alterations, attenuated pathological changes and alleviated renal tubular injury. Artemether also downregulated TGF-β1 mRNA expression levels, inhibited tubular proliferation, restored tubular cell phenotypes and suppressed proliferation-related signalling pathways. In addition, artemether restored renal redox imbalance, increased mtDNA copy number and improved mitochondrial function. In summary, we provided initial evidence that artemether ameliorates kidney injury by restoring redox imbalance and improving mitochondrial function in Adriamycin nephropathy in mice. Artemether may be a promising agent for the treatment kidney disease.

## Introduction

Chronic kidney disease (CKD) is becoming a global health burden and is characterized by urine abnormalities, morphological structure alterations and impaired renal function^1^. It is urgent to explore the pathogenesis of CKD and develop effective drugs retarding its progression.

As an organ with high energy demand, the kidney is rich in mitochondria especially in renal tubular cells. Mitochondria are considered the cell’s powerhouse and participate in many cellular processes. Emerging evidence suggests that mitochondrial dysfunction plays a pivotal role in kidney injury^2^. In a community based population, a negative association between mitochondrial DNA (mtDNA) copy number in peripheral blood and the prevalence of microalbuminuria was observed^3^. A urinary excretion metabolomics study and renal gene expression analysis revealed that citric acid cycle activity is impaired in CKD patients^4^. Mitochondria are the major site of fatty acid oxidation. In humans and mouse models with tubulointerstitial fibrosis, defective fatty acid oxidation was detected. Restoring fatty acid metabolism using genetic or pharmacological methods significantly prevented the progression of kidney fibrosis^5^.

Oxidative stress is defined as an imbalance in the generation of reactive oxygen species (ROS) in excess of the capacity of cells/tissues to detoxify or scavenge them. Numerous studies have demonstrated that dysregulation of the kidney redox state promotes fibrogenic pathways and eventually leads to renal failure^6^. Mitochondria are a major source of cellular ROS and regulate redox balance in cooperation with other organelles^7^. Redox imbalance, mitochondrial dysfunction and kidney injury are interconnected. However, the molecular mechanisms underlying these factors remain elusive.

Artemether is a derivative of artemisinin with improved bioavailability and is widely used in antimalarial treatment^8,9^. Previous studies have shown that artemisinin derivatives have antiviral, antifungal, anticancer, and antidiabetic activities^10–15^. Several studies have demonstrated that the biological effects of artemether are related to the regulation of mitochondria^16–18^. However, the precise mechanisms are not completely understood.

In this study, we explored the role of artemether in Adriamycin nephropathy (AN) and investigated the underlying mechanisms.

## Results

### Artemether reduced urinary protein excretion and recovered podocyte alterations

**Figure 1:**
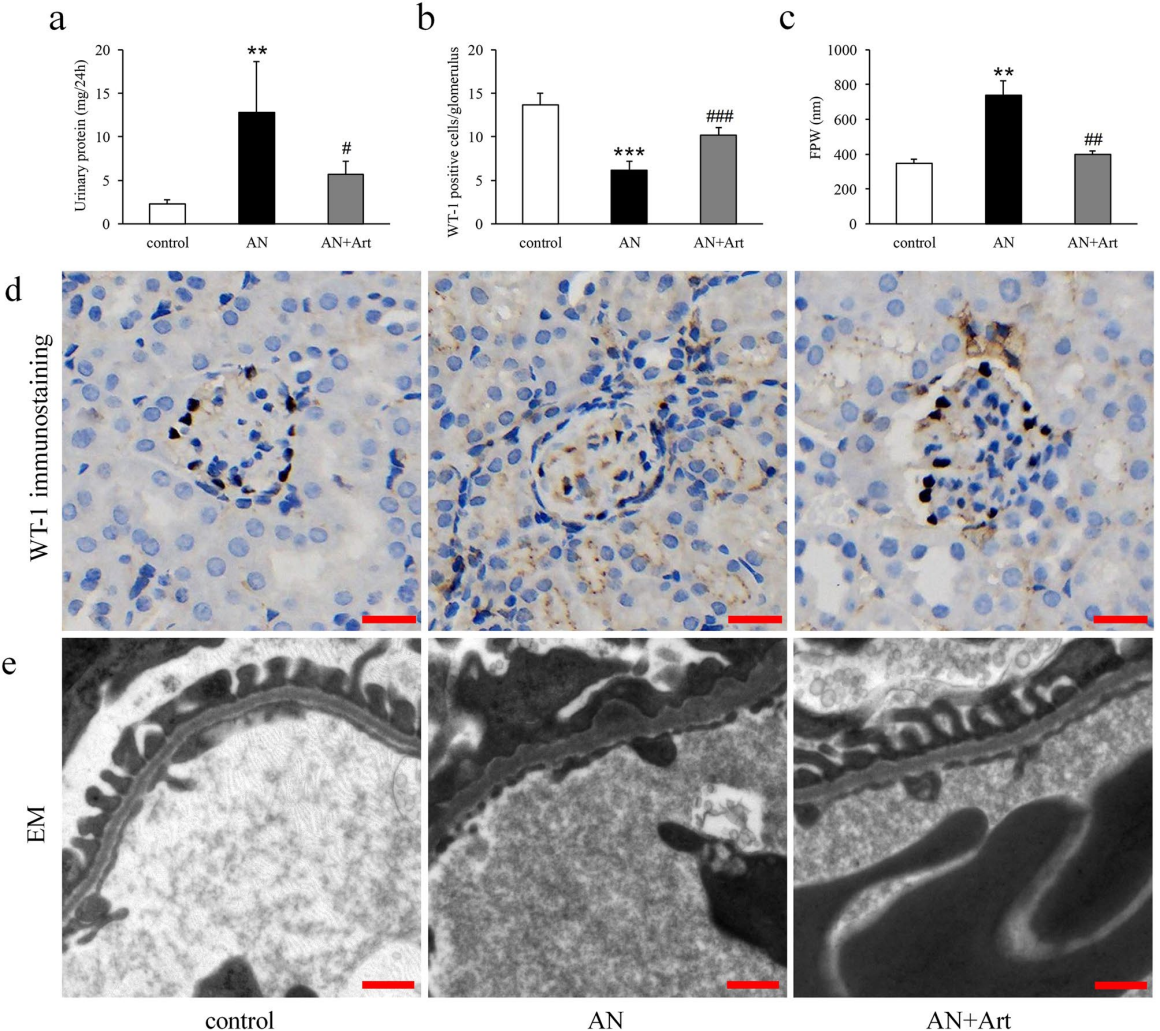
Artemether reduced urinary protein excretion and recovered podocyte alterations. (**a**) Quantification and statistical analysis of urinary protein excretion in various groups. n = 6 per group. (**b**) Counting of WT-1 positive cells per glomerulus in each group. n = 6 per group. (**c**) Measurement of FPW in different groups. n = 3 per group. (**d**) Representative images of WT-1 immunostaining in each group. Scale bar, 20 μm. (**e**) Representative TEM images displaying morphological changes of foot processes. Scale bar, 500 nm. **P < 0.01 and ***P < 0.001 versus control; ^#^*P* < 0.05, ^##^*P* < 0.01 and ^###^*P* < 0.001 versus AN.

Proteinuria is a marker of renal injury and an independent risk factor for the progression of CKD. As shown in Fig. 1a, compared to the control group, urinary protein excretion in the AN group increased significantly and was reduced significantly by artemether treatment. Podocytes play an important role in proteinuria formation. Therefore, we counted the number of WT-1 positive cells (indicating podocyte number) and measured foot process width (FPW) in the glomerulus. The results revealed that podocyte number decreased and FPW increased significantly in AN group mice (Fig. 1b–e). Artemether treatment obviously recovered these podocyte alterations (Fig. 1b–e).

### Artemether attenuated pathological changes and renal tubular injury

**Figure 2:**
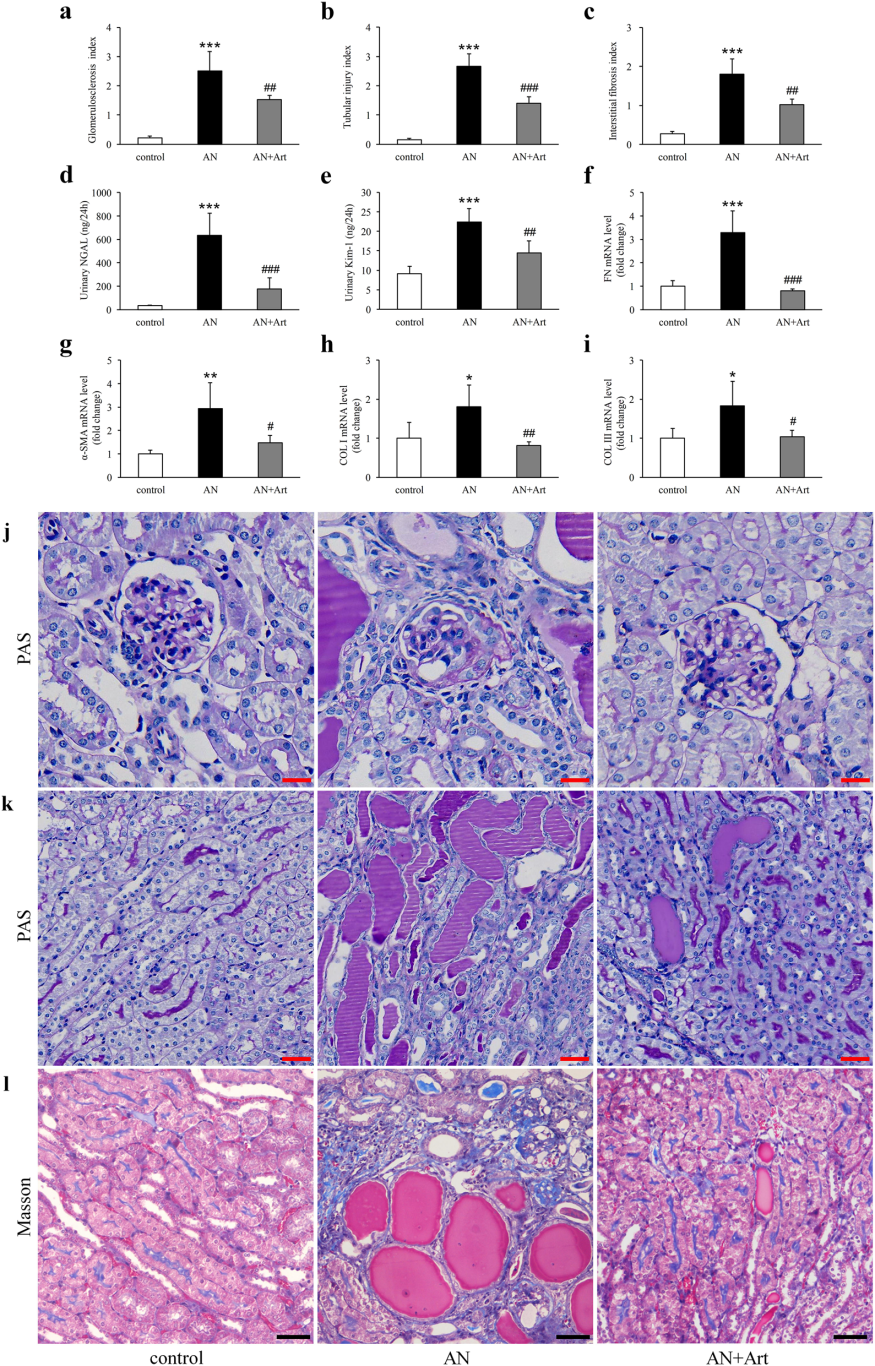
Artemether attenuated pathological changes and renal tubular injury. Evaluation of (**a**) glomerulosclerosis, (**b**) tubular injury, and (**c**) interstitial fibrosis in each group. Quantification of urinary (**d**) NGAL and (**e**) Kim-1 excretion in various groups. Determination of renal mRNA expression levels of (**f**) FN, (**g**) α-SMA, (**h**) COL I, and (**i**) COL III in different groups by qPCR and normalization against the housekeeping gene β-actin. (**j**, **k**) Representative images of PAS staining for glomeruli (scale bar, 20 μm) and tubules (scale bar, 50 μm). (**l**) Representative images of Masson trichrome staining for renal tubulointerstitium. Scale bar, 50 μm. n = 6 per group. *P < 0.05, **P < 0.01 and ***P < 0.001 versus control; ^#^*P* < 0.05, ^##^*P* < 0.01 and ^###^*P* < 0.001 versus AN.

At the end of this study, focal segmental glomerulosclerosis (Fig. 2a,j), tubular injury (Fig. 2b,k), and tubulointerstitial fibrosis (Fig. 2c,l) were prominent in AN group mice. Artemether treatment significantly ameliorated these pathological lesions (Fig. 2a–c,j–l). Consistent with the tubular pathological changes, urinary Kim-1 and NGAL levels, which are two tubular injury biomarkers, increased significantly in the AN group and were significantly reduced by artemether (Fig. 2d,e). In addition, the fibrosis-related mRNA (fibronectin: FN, α-smooth muscle actin: α-SMA, collagen: COL I, and COL III) levels also increased significantly in AN group mice and were downregulated by artemether treatment (Fig. 2f–i). Consistent with these molecular and pathological changes, the increased serum creatinine and BUN in AN group were significantly reduced by artemether (Fig. 9a,b).

### Artemether inhibited tubular cell proliferation

**Figure 3:**
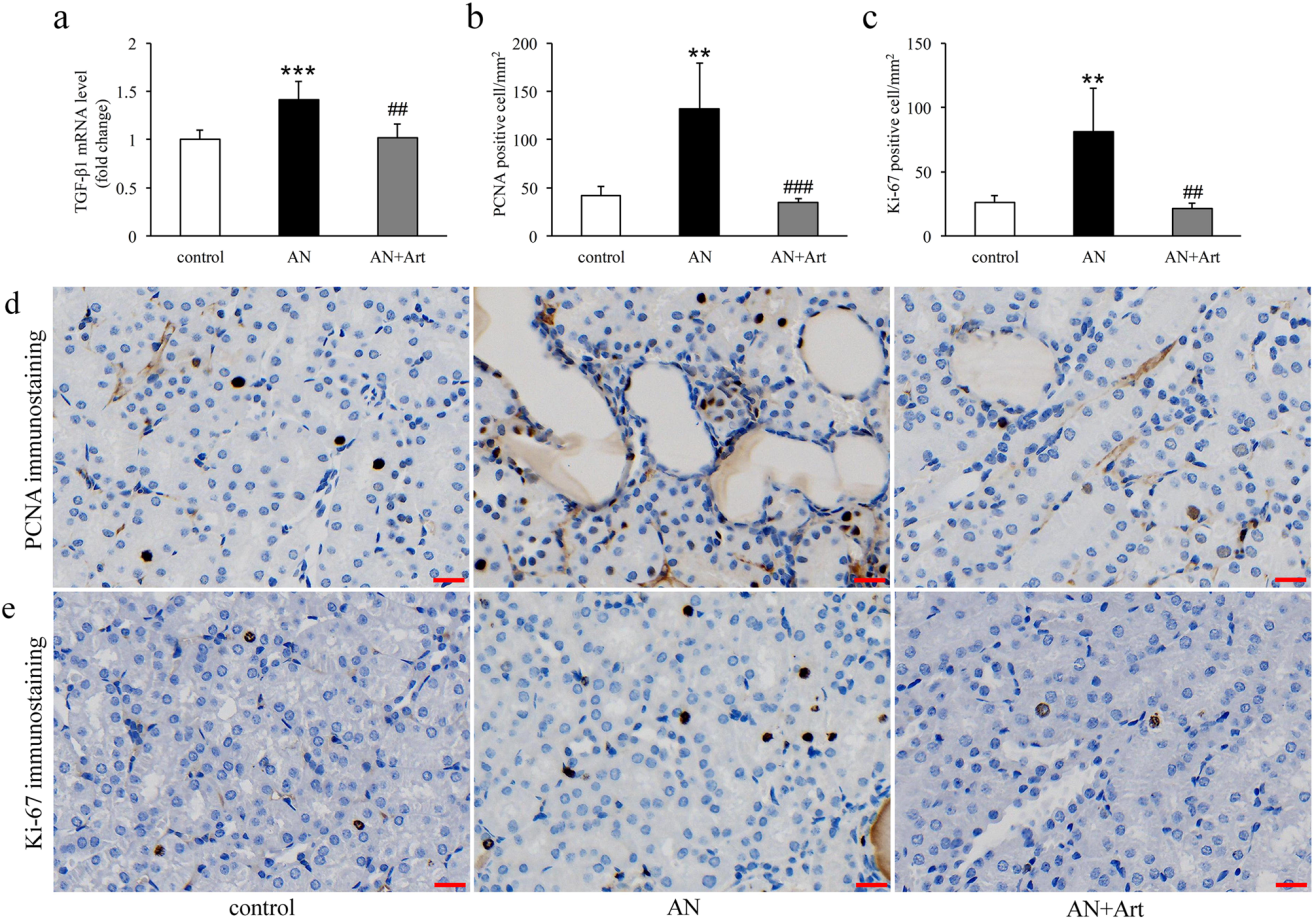
Artemether downregulated TGF-β1 mRNA expression levels and inhibited tubular proliferation. (**a**) Determination of renal TGF-β1 mRNA expression levels in each group. (**b**, **c**) Counting of PCNA and Ki-67 positive tubular cells per mm^2^ in various groups. (**d**, **e**) Representative images of PCNA and Ki-67 immunostaining in each group. Scale bar, 20 μm. n = 6 per group. **P < 0.01 and ***P < 0.001 versus control; ^##^*P* < 0.01 and ^###^*P* < 0.001 versus AN.

To determine the state of cell growth in the kidney, we performed immunohistochemistry for the cell proliferation markers proliferating cell nuclear antigen (PCNA) and Ki-67. The results revealed that PCNA and Ki-67 positive tubular cells increased significantly in AN group mice and were significantly decreased by artemether (Fig. 3b–e). TGF-β1 is a critical regulator of cell proliferation. Compared to control group mice, marked upregulation of TGF-β1 mRNA in renal tissue was observed in AN group mice, and it was significantly downregulated by artemether treatment (Fig. 3a).

### Artemether restored the tubular cell phenotype and suppressed proliferation-related signalling pathways

**Figure 4:**
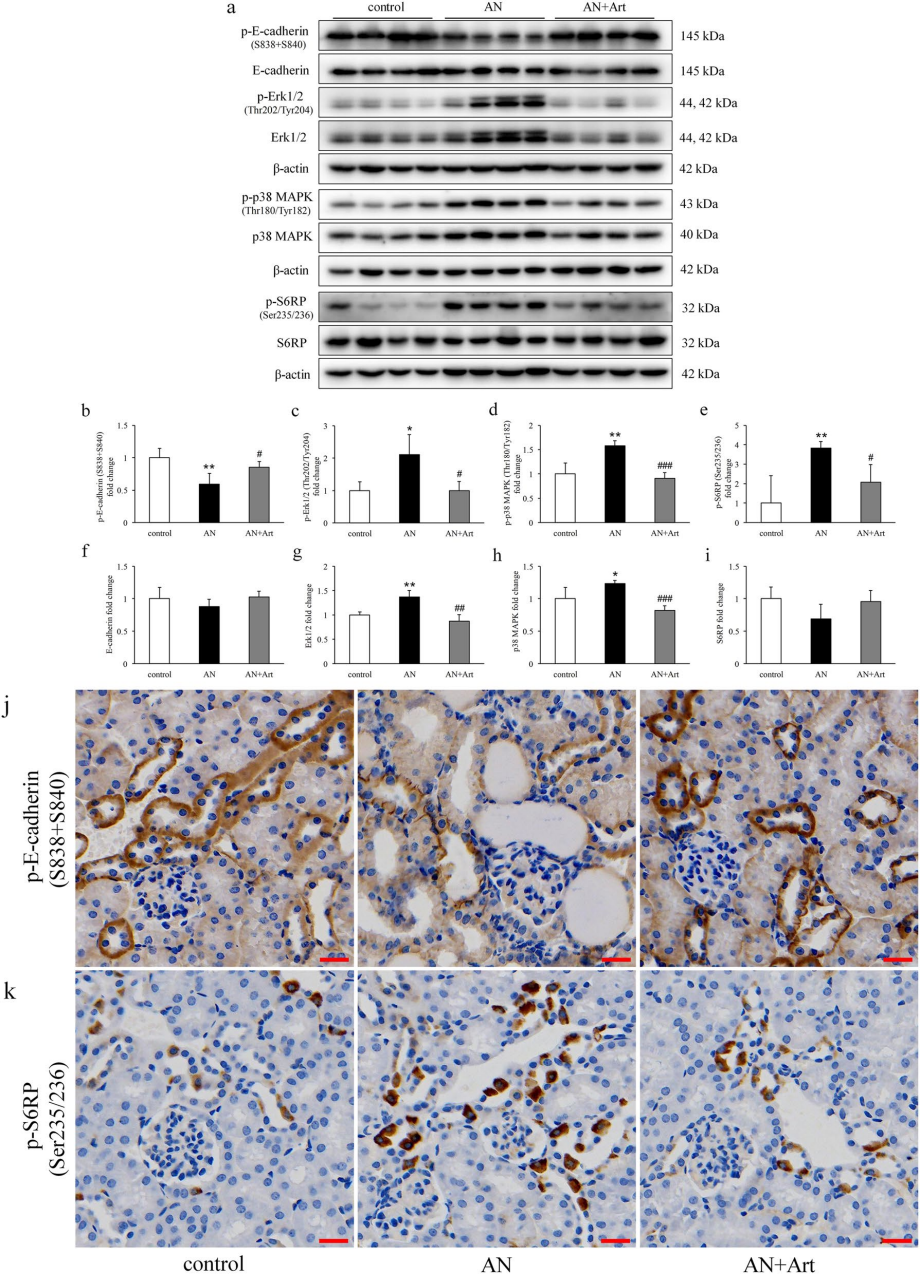
Effects of artemether on tubular cell phenotype and proliferation-related signalling pathways. (**a**–**i**) Western blot bands and quantitative analysis of p-E-cadherin (S838 + S840), E-cadherin, p-Erk1/2 (Thr202/Tyr204), Erk1/2, p-p38 MAPK (Thr180/Tyr182), p38 MAPK, p-S6RP (Ser235/236), and S6RP in kidney in each group. (**j**, **k**) Representative immunohistochemical staining images of p-E-cadherin (S838 + S840) and p-S6RP (Ser235/236) in the kidney. Images showed that these proteins are predominantly expressed in renal tubules. Scale bar, 20 μm. n = 6 per group. *P < 0.05 and **P < 0.01 versus control; ^#^*P* < 0.05, ^##^*P* < 0.01 and ^###^*P* < 0.001 versus AN.

**Figure 5:**
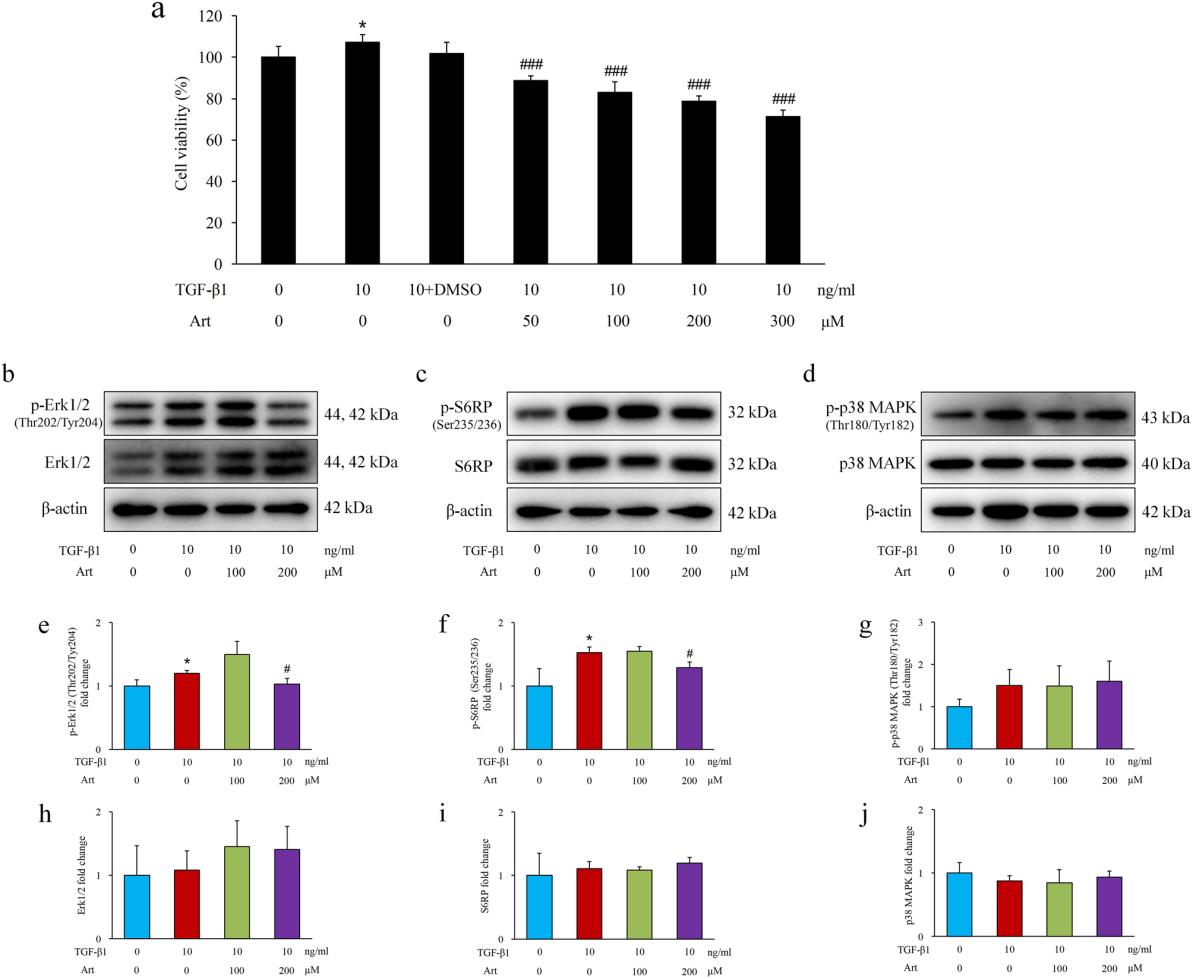
Effects of artemether on cell viability and proliferation-related signalling pathways in vitro. (**a**) Cell viability was analysed by MTT assay after treatment with TGF-β1 and different concentrations of artemether for 24 h. n = 6 per group. *P < 0.05 vs. TGF-β1:0 + Art: 0 group; ^###^*P* < 0.001 vs. TGF-β1:(10+DMSO) + Art: 0 group. (**b**–**d**) Representative western blot bands of p-Erk1/2 (Thr202/Tyr204), Erk1/2, p-S6RP (Ser235/236), S6RP, p-p38 MAPK (Thr180/Tyr182), and p38 MAPK after treatment with the indicated amount of artemether and TGF-β1. (**e**–**j**) Bar graphs presenting the fold change of these proteins after normalization to β-actin. n = 3 per group. *P < 0.05 versus TGF-β1:0 + Art: 0 group; ^#^*P* < 0.05 versus TGF-β1:10 + Art: 0 group.

As a representative epithelial phenotype marker, E-cadherin (phospho S838 + S840) was reduced significantly in the AN group and was upregulated by artemether (Fig. 4a,b,f,j). The proliferation-related signals Erk1/2, p38, and S6RP were activated in AN group mice and were inhibited by artemether (Fig. 4a,c–e,g–i,k). To determine whether the effects of artemether on tubular cells were mediated by TGF-β1, we performed an in vitro study. NRK-52E cells were incubated with 10 ng/ml TGF-β1 in combination with or without artemether for 24 h. Compared to the untreated group, TGF-β1 significantly increased cell viability (Fig. 5a) and induced Erk1/2 and S6RP activation (Fig. 5b,e,h,c,f,i). The coincubation of artemether dose-dependently decreased cell viability (Fig. 5a) and inhibited Erk1/2 and S6RP activation at a concentration of 200 μM (Fig. 5b,e,h,c,f,i). The p38 signal was slightly activated by TGF-β1 stimulation but not to a significant degree. Artemether intervention also had no effect on p38 signalling (Fig. 5d,g,j).

### Effects of artemether on kidney redox balance

**Figure 6:**
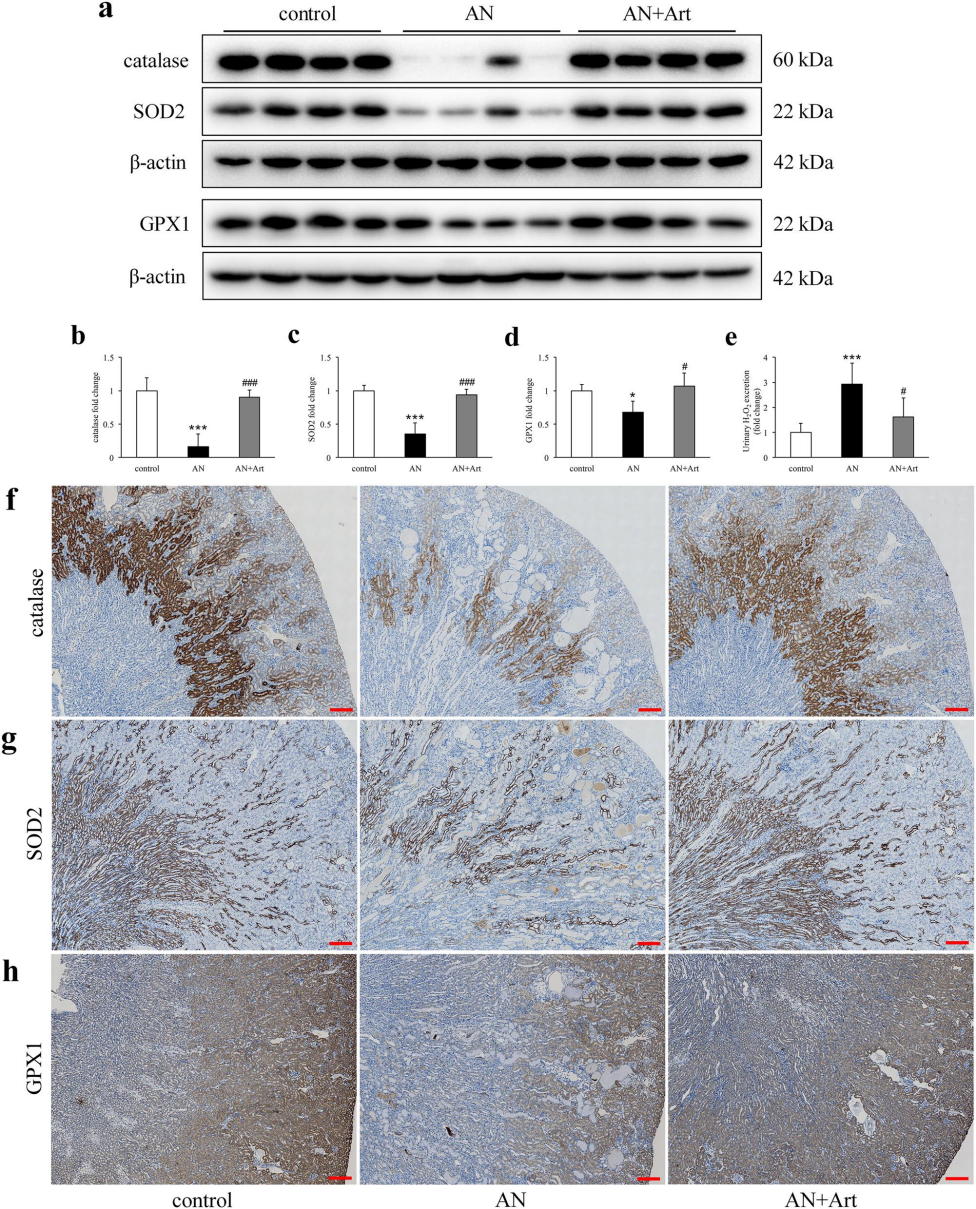
Effects of artemether on kidney redox balance and urinary H_2_O_2_ excretion. (**a**) Western blot images displaying renal protein levels of catalase, SOD2, and GPX1 in each group. (**b**–**d**) Bar graphs showing the fold change of these proteins after normalization to β-actin. n = 4 per group. (**e**) Urinary H_2_O_2_ excretion in various groups. n = 6 per group. (**f**–**h**) Representative immunostaining images of renal catalase, SOD2, and GPX1 in each group. Scale bar, 200 μm. *P < 0.05 and ***P < 0.001 versus control; ^#^*P* < 0.05 and ^###^*P* < 0.001 versus AN.

Compared to the control group, the redox associated enzymes including catalase, superoxide dismutase 2 (SOD2), and glutathione peroxidase 1 (GPX1), were all reduced significantly in the AN group and were significantly increased by artemether treatment (Fig. 6a–d). Immunohistochemical staining revealed that these enzymes were mainly expressed in renal tubules and rarely expressed in glomeruli. Their distribution in tubules also exhibited a specific pattern. Catalase was mainly expressed in the cortico-medullary junction, SOD2 was mainly expressed in the medulla, and GPX1 was mainly expressed in the cortex (Fig. 6f–h). In line with the redox imbalance in the kidney, urinary H_2_O_2_ excretion increased significantly in the AN group and was reduced by artemether (Fig. 6e).

### Artemether increased mitochondrial mass and improved mitochondrial function

**Figure 7:**
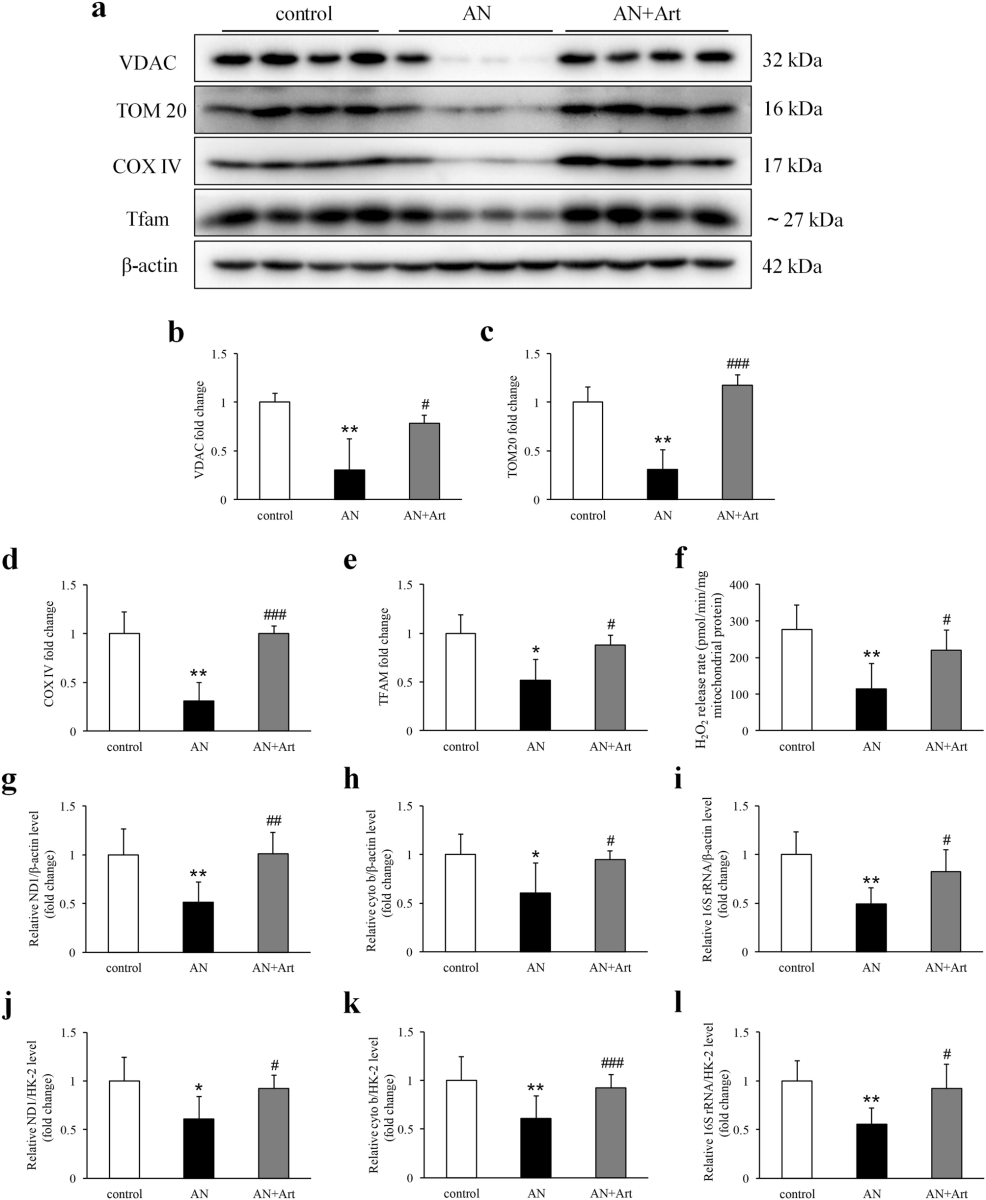
Artemether increased mitochondrial mass and improved mitochondrial function. (**a**–**e**) Western blot images and quantitative analysis of VDAC, TOM20, COX IV, and Tfam in renal tissue of various groups. n = 4 per group. (**f**) Mitochondrial H_2_O_2_ release rate in each group after artemether treatment for 2 weeks. n = 6 per group. (**g**–**l**) Bar graphs indicating the quantification of mitochondrial DNA copy number (determined by qPCR for ND1, cytochrome b, and 16S rRNA against β-actin and HK-2) in each group. n = 6 per group. *P < 0.05 and **P < 0.01 versus control; ^#^*P* < 0.05, ^##^*P* < 0.01 and ^###^*P* < 0.001 versus AN.

In AN group mice, loss of voltage-dependent anion channel (VDAC), translocase of the outer mitochondrial membrane 20 (TOM20), cytochrome c oxidase IV (COX IV), and mitochondrial transcription factor A (Tfam) were observed and these effects were increased by artemether treatment (Fig. 7a–e). Because Tfam protein plays an important role in the maintenance mtDNA copy number, we measured mtDNA copy numbers in each group. Consistent with the reduction in Tfam, mtDNA copy number was also reduced in the AN group and was increased by artemether (Fig. 7g–l). In addition, a lower renal mitochondrial H_2_O_2_ release rate was observed in the AN group, which was increased by artemether treatment (Fig. 7f).

### Artemether regulated proton leak and AMPK signalling

**Figure 8:**
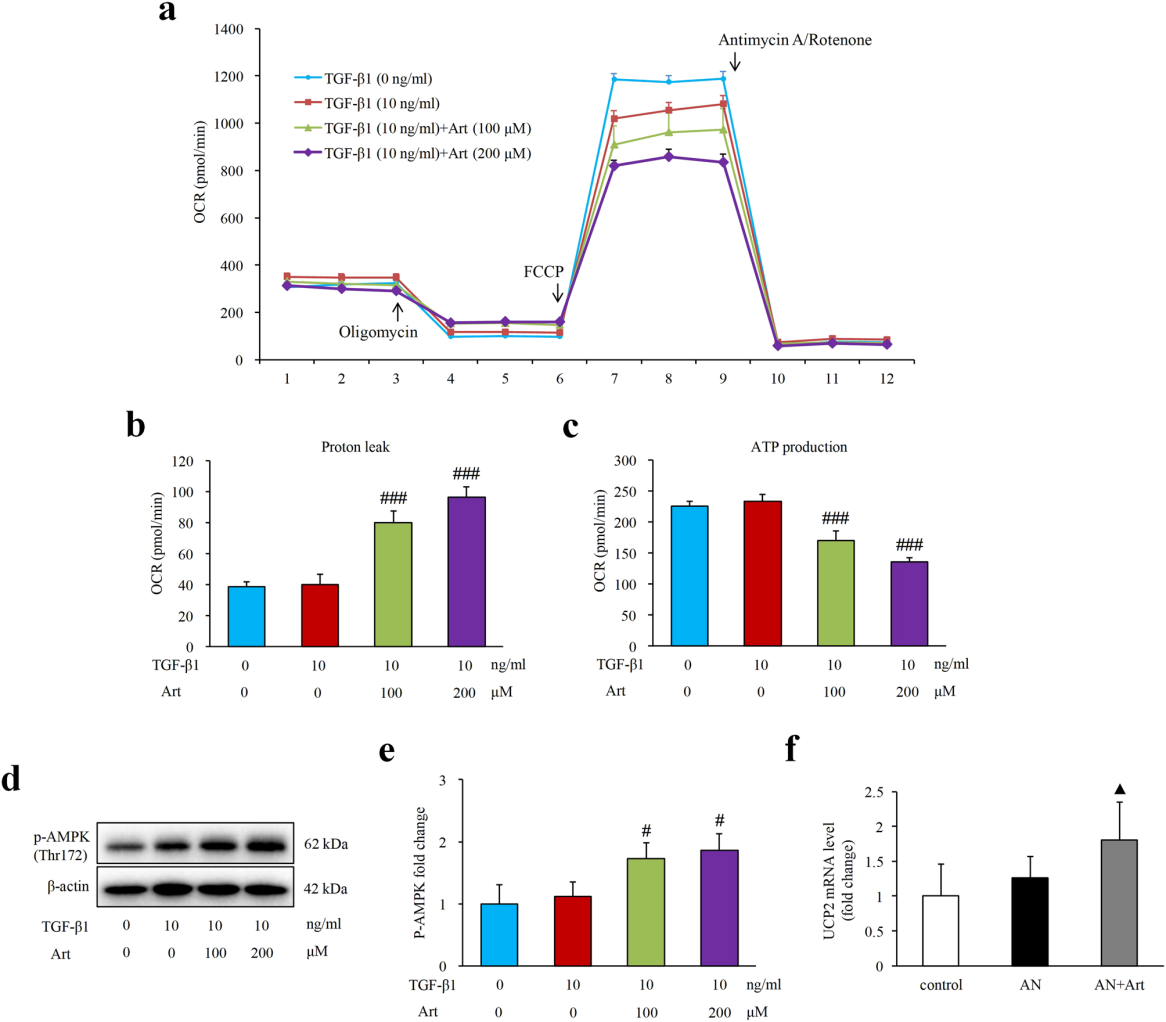
Effects of artemether on proton leak and AMPK signalling. (**a**) Cellular mitochondrial respiration was measured by a Seahorse XF24 extracellular flux analyser after cells were treated with the indicated amount of artemether and TGF-β1 for 24 h. (**b**, **c**) Bar graphs presenting the calculation of proton leak and ATP production in various groups according the manufacturer’s protocol. n = 5 per group. (**d**, **e**) Representative western blot images and quantitative analysis of p-AMPK (Thr172) fold change after TGF-β1 and artemether treatment for 24 h. n = 3 per group. (**f**) Renal mRNA expression level of UCP2 in each group mice normalized against the housekeeping gene β-actin. n = 6 per group. ^#^*P* < 0.05 and ^###^*P* < 0.001 versus TGF-β1:10 + Art: 0 group; ^▲^*P* < 0.05 versus control.

To explore the direct effect of artemether on mitochondria, we performed a mitochondrial stress test using the Seahorse XF analyser. Compared to the untreated group, TGF-β1 stimulation did not affect proton leak or ATP production (Fig. 8a–c). In contrast, artemether treatment significantly increased proton leak and decreased ATP production (Fig. 8a–c). As shown in Fig. 8d,e, the AMPK signal was activated by artemether. Moreover, the in vivo study showed that uncoupling protein (UCP2) mRNA levels in the kidney were significantly upregulated by artemether (Fig. 8f).

**Figure 9:**
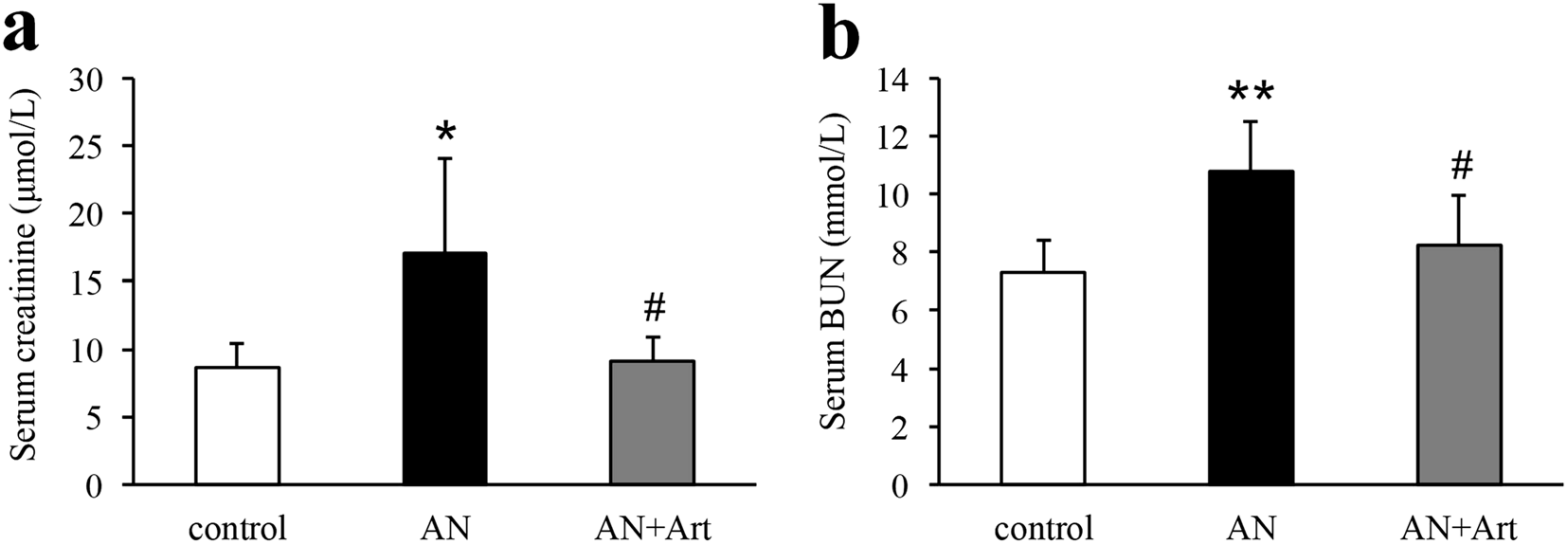
The levels of (**a**) serum creatinine and (**b**) BUN in each group. n = 6 per group. *P < 0.05 and **P < 0.01 versus control; ^#^*P* < 0.05 versus AN.

## Discussion

Accumulating studies have highlighted the important role of mitochondria in kidney disease, but the detailed mechanisms remain largely unknown^19^. In this study, we found that artemether improved kidney injury in Adriamycin-induced nephropathy in mice by regulating mitochondrial proton leak.

It is well recognized that proteinuria is a risk factor for the progression of CKD. In the current study, artemether reduced urinary protein excretion, attenuated podocyte loss and ameliorated foot process fusion. Renal fibrosis is the common pathway of various CKD. It involves an excess accumulation of extracellular matrix which is primarily composed of FN, α-SMA and collagen^20^. Artemether obviously attenuated glomerulosclerosis and interstitial fibrosis and downregulated the mRNA expression levels of FN, α-SMA, COL I and COL III in the kidney. These results suggest that artemether has potent antifibrotic effects. In addition, artemether significantly ameliorated tubular pathological injury and reduced urinary NGAL and Kim-1 excretion, which are tubular injury biomarkers. The renal tubule has long been regarded as the victim of kidney injury. However, an increasing number of studies have demonstrated that renal tubules are the primary sensor and driving force in kidney disease progression^21^.

Under normal conditions, renal tubular cells divide at a low rate as evaluated by PCNA and Ki-67 immunoreactivity. In the stressed kidney after Adriamycin injection, tubular cells presented a hyperproliferative state. Their phenotype was also changed, which was verified by the loss of p-E-cadherin. Consistent with the hyperproliferation state, multiple growth and proliferation-related signalling pathways, including Erk1/2, p38 MAPK and S6RP, were activated in AN group mice. Previous studies have revealed that TGF-β1 plays a key role in phenotype alteration and tubulointerstitial fibrosis^22^. In mice with Adriamycin-induced nephropathy, TGF-β1 mRNA expression in the kidney increased significantly, which explained the pathological changes that occurred in tubular cells. Artemether treatment evidently inhibited tubular cell proliferation, restored p-E-cadherin expression, and downregulated TGF-β1 mRNA levels. Therefore, we hypothesized that the effects of artemether on tubular cells may be associated with TGF-β1. To test this hypothesis, we performed an in vitro study, and the results indicated that TGF-β1 could increase cell viability and induce Erk1/2 and S6RP activation after 24-h incubation with NRK-52E cells. The coincubation of artemether dose-dependently decreased cell viability and inhibited Erk1/2 and S6RP activation at a concentration of 200 μM. However, p38 MAPK activation did not reach a significant degree after TGF-β1 stimulation, and artemether intervention had no inhibitory effect on p38 MAPK activation. This discrepancy with the in vivo study might be associated with different drug doses and intervention times.

Another interesting finding in this experiment is that the distribution of catalase, SOD2, and GPX1 exhibited spatial specificity in the kidney. All of these proteins were mainly expressed in renal tubules and rarely expressed in glomeruli. Reduction of these ROS scavengers and increased urinary H_2_O_2_ excretion were observed in AN group mice. The redox imbalance was well restored by artemether. It is generally accepted that mitochondria have been implicated in the maintenance of redox homeostasis and cell proliferation^23^. Therefore, we hypothesized that artemether may target mitochondria and then exert its biological activities. Renal tubules are packed with mitochondria and particularly vulnerable to a variety of injuries, including proteinuria, toxins, hypoxia, obstruction and metabolic disorders^21^. In Adriamycin nephropathy, tubular loss and injury are the common pathological lesions. Consistent with the loss of normal renal histological structure, mitochondrial components, including VDAC, TOM20, and COX IV, decreased remarkably in AN group mice. In addition, significantly reduced Tfam and mtDNA copy numbers were also detected. As we expected, mitochondrial content and mtDNA were obviously elevated by artemether. The conventional viewpoint noted that H_2_O_2_ generation is a by-product of mitochondrial metabolism^24^. However, growing research has revealed that H_2_O_2_ serves as an important signalling molecule, and its production can be regarded as an indicator of healthy mitochondria and physiological oxidative phosphorylation^25,26^. In the present study, a lower renal mitochondrial H_2_O_2_ release rate was measured in AN group mice, implying that mitochondria were compromised. Similarly, artemether greatly improved the impaired mitochondrial function.

In the current study, another important finding was that artemether could induce mitochondrial proton leak and reduce ATP production. In response to the uncoupling effect of artemether, the cellular energy status sensor AMPK was also activated. In addition, the in vivo study revealed that artemether could upregulate the UCP2 mRNA levels in the kidney. These clues together imply that artemether may be involved in regulating mitochondrial energy metabolism. However, the detailed mechanism still needs to be further explored.

In summary, our study demonstrated that artemether could ameliorate kidney injury in Adriamycin nephropathy in mice by regulating mitochondrial proton leak. The findings strongly suggest that artemether might be a useful pharmaceutical agent for treating kidney disease.

## Methods

### Reagents

Adriamycin was purchased from Sigma-Aldrich (St. Louis, MO, USA). Artemether was obtained from ConBon Biotechnology (Chengdu, Sichuan, China). Antibodies against WT-1, PCNA, Ki-67, and HRP-polymer conjugated anti-Mouse/Rabbit IgG complex were provided by MaiXin Biotechnology (Fuzhou, Fujian, China). Antibodies against p-E-cadherin (S838 + S840) and E-cadherin were purchased from Abcam (Cambridge, UK). Antibodies against p-Erk1/2 (Thr202/Tyr204), Erk1/2, p-p38 MAPK (Thr180/Tyr182), p38 MAPK, p-S6RP (Ser235/236), S6RP, catalase, SOD2, VDAC, TOM20, COX IV, and p-AMPK (Thr172) were purchased from Cell Signaling Technology (Danvers, MA, USA). The GPX1 antibody was obtained from GeneTex (Irvine, CA, USA). The Tfam antibody was obtained from Novus Biologicals (Littleton, CO, USA), and the β-actin antibody was purchased from Sigma-Aldrich. Horseradish peroxidase (HRP)-conjugated secondary antibody was provided by Invitrogen (Carlsbad, CA, USA). Dulbecco’s modified Eagle’s medium (DMEM), fetal bovine serum (FBS), trypsin solution (EDTA), sodium pyruvate, and penicillin/streptomycin were purchased from Gibco (Invitrogen). L-glutamine, glucose, and dimethyl sulfoxide (DMSO) were obtained from Sigma-Aldrich.

### Animals

Male BALB/c mice (weighing 20–25 g) were provided by the Laboratory Animal Center of Southern Medical University (Guangzhou, China) and housed in the Central Animal Facility at Shenzhen Graduate School of Peking University. Adriamycin nephropathy (AN) was established by a single injection of Adriamycin (10.4 mg/kg) via the tail vein. Then, they were randomly allocated into the AN group (n = 6) and AN + artemether (AN + Art) group (n = 6). Control group (n = 6) mice were injected with the same volume of normal saline. Two weeks after Adriamycin injection, AN + Art group mice were fed a regular diet supplemented with 0.3 g/kg artemether. Control and AN group mice were fed a regular diet. The treatment lasted for 2 weeks. All animal procedures were approved by the Guangzhou University of Chinese Medicine Institutional Animal Care and Use Committee. All experiments were performed in accordance with relevant guidelines and regulations.

### Biochemical determination

The serum creatinine and blood urea nitrogen (BUN) levels were determined by using an automatic biochemical analyzer (Roche, Basel, Switzerland).

### Cell culture and treatment

Normal rat kidney epithelial cells (NRK-52E) were obtained from American Type Culture Collection (ATCC, Manassas, VA, USA). Cells were cultured in DMEM supplemented with 10% FBS, 5.5 mM glucose, 4 mM l-glutamine, 1 mM sodium pyruvate and 1% penicillin/streptomycin. Cells were maintained in a humidified incubator at 37 °C with 5% CO_2_. When 80% confluence was reached, cells were switched to medium containing the indicated amount of artemether and TGF-β1 (10 ng/ml, R&D Systems, Minneapolis, MN, USA) for 24 h.

### MTT assay

Cell viability was determined by MTT assay. Briefly, NRK-52E cells (100 μl, 2 × 10^5^ cells/ml) were seeded into 96-well culture plates and incubated for 24 h to allow cells to attach. The cells were treated with different concentrations of artemether containing TGF-β1 (10 ng/ml) for 24 h. Then the medium was removed and incubated with 20 μl MTT solution (5 mg/ml) for 4 h. After incubation, the medium was replaced with 150 μl DMSO and gently shaken for 10 min. The absorbance was detected at 570 nm by using Synergy H1 microplate reader (BioTek Instruments, Winooski, VT, USA).

### Oxygen consumption rate

The cellular oxygen consumption rate (OCR) was measured by using the Seahorse XF24 extracellular flux analyser (Agilent Technologies, Santa Clara, CA, USA). NRK-52E cells (60 × 10^3^ cells per well) were seeded into 24-well microplates and treated with the indicated amount of artemether and TGF-β1. After 24 h of incubation, the medium was replaced with seahorse buffer, and then oligomycin, FCCP, and rotenone/antimycin A were automatically injected into the assay medium to bring the final concentration to 1 μmol/L, 2 μmol/L, and 0.5 μmol/L. OCR values were calculated according to the manufacturer’s protocol.

### Histological analysis

The mice were sacrificed 2 weeks after artemether treatment (4 weeks after Adriamycin injection). Paraffin-embedded renal sections (4 μm) were stained with periodic acid-Schiff (PAS) and Masson trichrome. Thirty glomeruli and twenty tubular areas in each section were randomly selected to evaluate renal glomerulosclerosis and tubular injury. The index was scored on a scale of 0 to 4 (0, < 5%; 1, 5–25%; 2, 25–50%; 3, 50–75%; 4, > 75%) based on PAS staining as previously described^27^. Masson trichrome staining was used to evaluate interstitial fibrosis which was defined as the area occupied by positive interstitium. Fifteen randomly selected fields in the cortex were evaluated for each section. The degree of interstitial fibrosis index was graded on a scale of 0 to 4 (0, < 5%; 1, 5–25%; 2, 25–50%; 3, 50–75%; 4, > 75%).

### Transmission electron microscopy

The renal cortex (1 mm^3^ sample) was fixed in 2.5% glutaraldehyde and then postfixed in 1% osmic acid for transmission electron microscopy (TEM). The TEM images were photographed by JEM-1400 (JEOL, Tokyo, Japan). Eight images in each sample were selected to measure the FPW by using ImageJ software (National Institutes of Health, Bethesda, MD, USA). The average podocyte FPW was calculated using a previously described method^28^.

### Immunohistochemistry

Immunohistochemical staining was performed on 4 μm thick renal sections. After antigen retrieval with citrate buffer, sections were incubated with primary antibodies against WT-1, PCNA, Ki-67, p-E-cadherin (S838 + S840), p-S6RP (Ser235/236), catalase, SOD2, and GPX1. Then the sections were washed and incubated with HRP-polymer conjugated anti-Mouse/Rabbit IgG complex. Localization of peroxidase conjugates was detected using diaminobenzidine tetrahydrochloride solution as chromogen and counterstained with haematoxylin.

### ELISA

Urinary NGAL and Kim-1 (R&D Systems, Minneapolis, MN, USA) levels were measured by ELISA kit according to the manufacturer’s instructions.

### Urinary protein and H_2_O_2_ assay

The urinary protein was detected by using the Bio-Rad protein assay (Bio-Rad Laboratories, Hercules, CA, USA). Urinary H_2_O_2_ was determined by using Amplex UltraRed reagent (Invitrogen) according to the manufacturer’s instructions.

### Mitochondrial H_2_O_2_ release rate

Kidney mitochondria were isolated from each group of mice as previously described^29^. The mitochondrial H_2_O_2_ release rate was detected by Amplex UltraRed reagent according to the manufacturer’s instructions. Briefly, the same amount of mitochondrial proteins in each group was added to the mitochondrial assay medium which was preadded to the microplate wells. Then, Amplex UltraRed/HRP working solution was added to initiate the reaction. The fluorescence was detected at Ex/Em 490/585 nm using a Synergy H1 microplate reader.

### Immunoblotting analysis

Samples were prepared in sample loading buffer (Bio-Rad). The lysates were separated on SDS-PAGE gels and transferred to PVDF membranes (Merck Millipore, Danvers, MA, USA). After blocking, the membranes were incubated overnight with the following primary antibodies: p-E-cadherin (S838 + S840), E-cadherin, p-Erk1/2 (Thr202/Tyr204), Erk1/2, p-p38 MAPK (Thr180/Tyr182), p38 MAPK, p-S6RP (Ser235/236), S6RP, catalase, SOD2, GPX1, VDAC, TOM20, COX IV, Tfam, p-AMPK (Thr172), and β-actin. Then the membranes were incubated with secondary antibodies and detected using the ChemiDoc MP Imaging System (Bio-Rad).

### Gene expression analysis and mtDNA copy number measurement

Total RNA extraction was performed using the TRIzol Plus RNA purification kit (Invitrogen). First-strand cDNA was generated by oligo(dT)^12–18^ primers and M-MLV reverse transcriptase (Invitrogen) according to the manufacturer's protocol. Renal tissue genomic DNA (gDNA) was extracted using a gDNA purification kit (Magen, Guangzhou, China). The DNA concentrations were determined by a Synergy H1 microplate reader and adjusted to the same concentrations. Quantitative real-time PCR (qPCR) was subsequently performed using SYBR green master mix (Applied Biosystems, Foster City, CA, USA) and gene-specific primers in the Stratagene M × 3000P real-time PCR system (Agilent Technologies). The primers were synthesized by Sangon Biotechnology Company (Shanghai, China) and are listed in Table 1. The amplification conditions were 95 °C for 5 min followed by 45 cycles of 95 °C for 15 s, 55 °C for 15 s, and 72 °C for 20 s. The relative mtDNA copy number or mRNA expression was calculated by 2^−ΔΔ*CT*^ and normalized against the housekeeping gene (β-actin for mRNA expression; β-actin and HK-2 for mtDNA copy number).

**Table 1:** Sequences of the primers for qPCR.

Gene	Primer sequence (5′–3′)
Mouse FN	F: GCAGTGACCACCATTCCTG
	R: CCTGTCTTCTCTTTCGGGTTCA
Mouse α-SMA	F: GTGCTATGTCGCTCTGGACTTTGA
	R: ATGAAAGATGGCTGGAAGAGGGTC
Mouse COL I	F: CTGGAACAAATGGGCTCACTG
	R: CAGGCTCACCAACAAGTCCTC
Mouse COL III	F: ACAGCTGGTGAACCTGGAAG
	R: ACCAGGAGATCCATCTCGAC
Mouse TGF-β1	F: CCGCAACAACGCCATCTATG
	R: CTCTGCACGGGACAGCAAT
Mouse ND1	F: CTAGCAGAAACAAACCGGGC
	R: CCGGCTGCGTATTCTACGTT
Mouse cyto b	F: GCCACCTTGACCCGATTCTTCGC
	R: TGAACGATTGCTAGGGCCGCG
Mouse 16S rRNA	F: CCGCAAGGGAAAGATGAAAGAC
	R: TCGTTTGGTTTCGGGGTTTC
Mouse HK2	F: GCCAGCCTCTCCTGATTTTAGTGT
	R: GGGAACACAAAAGACCTCTTCTGG
Mouse β-actin	F: GGACTCCTATGTGGGTGACG
	R: AGGTGTGGTGCCAGATCTTC
Mouse UCP2	F: GAGGTAGCAGGAAATCAGAATCATG
	R: TATCCAGAGGGAAAGTGATGAGATC

### Statistical analysis

All data are expressed as the mean ± SD. Data analysis was performed using SPSS statistics software (IBM, NY, USA). One-way analysis of variance (ANOVA) followed by Bonferroni post hoc analysis or unpaired Student’s t test was applied in the data analysis. Significance was defined as *P* < 0.05.

## Supplementary Information

Supplementary Information.

Supplementary Information.
