# Supplementary material for: Artemether ameliorates kidney injury by restoring redox imbalance and improving mitochondrial function in Adriamycin nephropathy in mice
Source: Sci Rep. 2021 Jan 14;11:1266. doi: 10.1038/s41598-020-80298-x (PMC7809108; doi:10.1038/s41598-020-80298-x)

**Methods**

**Biochemical determination.** The serum creatinine and blood urea nitrogen (BUN) levels were determined by using an automatic biochemical analyzer (Roche, Basel, Switzerland).

**Immunohistochemistry.** Immunohistochemical staining of cleaved caspase-3 and co-immunostaining of TOM20/4 HNE and WT-1 were performed on 4 μm thick renal sections. After antigen retrieval, sections were incubated with primary antibodies against cleaved caspase-3 (CST, Danvers, MA, USA), TOM20, 4 Hydroxynonenal (4 HNE, Abcam, Cambridge, UK), and WT-1 respectively. Then the sections were washed and incubated with horseradish peroxidase and alkaline phosphatase-labeled secondary antibodies. AP-Red (for TOM20, 4 HNE, and cleaved caspase-3) and diaminobenzidine (for WT-1) were used as chromogenic agents.

**Cell culture and treatment.** NRK-52E cells were seeded into 24-well culture plates and incubated for 24 hours to allow cells to attach. The cells were treated with different concentration of artemether containing adriamycin (1 μM) for 12 or 24 hours. Then the cells were prepared in sample loading buffer for immunoblotting analysis.

**Figure legends**

**Figure S1.** The levels of (a) serum creatinine and (b) BUN in each group. n=6 per group. **P*<0.05 and ***P*<0.01 vs. control; ^#^*P*<0.05 vs. AN.

**Figure S2.** Representative images of co-immunostaining of TOM20/4 HNE and WT-1 in each group. Scale bar, 10 μm for glomerulus; 40 μm for tubule.

**Figure S3.** Representative TEM images of mitochondria in podocyte in each group.

**Figure S4.** Representative images of immunohistochemical staining of cleaved caspase-3 in each group. Scale bar, 40 μm.

**Figure S5.** Western blots image (a) and quantitative analysis of (b) p-Erk1/2 (Thr202/Tyr204) in various groups. n=3 per group. ***P*<0.01 vs. Adriamycin: 0 + Art: 0 group; ^#^*P*<0.05 vs. Adriamycin: 1 + Art: 0 group.

**Figure S6.** Western blots images (a) and quantitative analysis of (b) SOD2 and (c) TOM20 in various groups. n=3 per group. ***P*<0.01 vs. Adriamycin: 0 + Art: 0 group; ^#^*P*<0.05 vs. Adriamycin: 1 + Art: 0 group.

**Figure S7.** Graphical scheme of the study (black arrow for adriamycin; red arrow for artemether).

**Figure S1-S7**

**Figure S1**


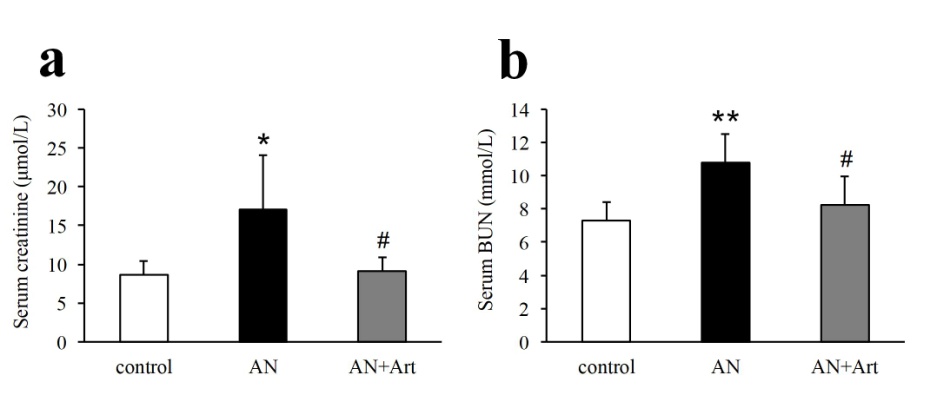


**Figure S2**


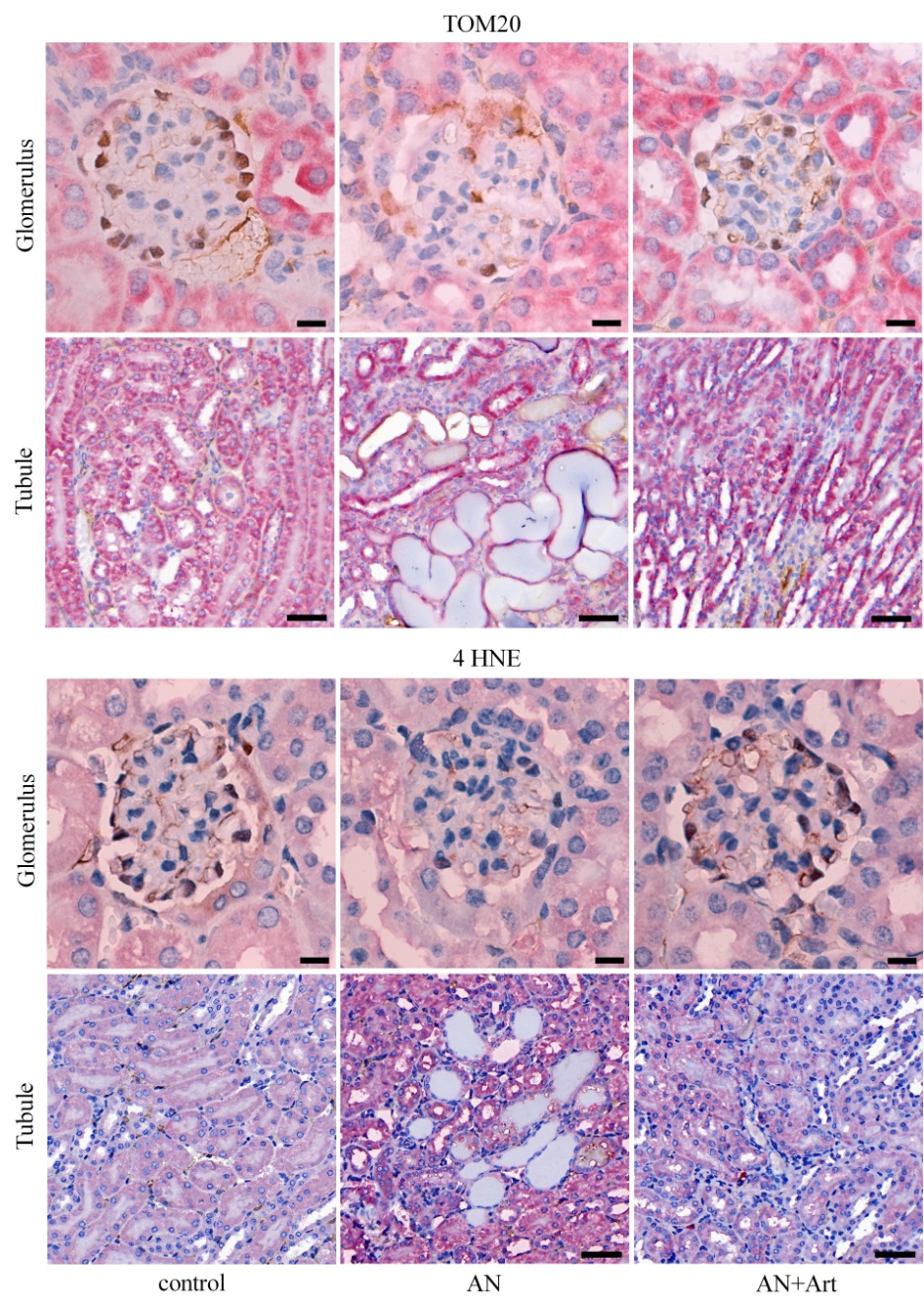


**Figure S3**


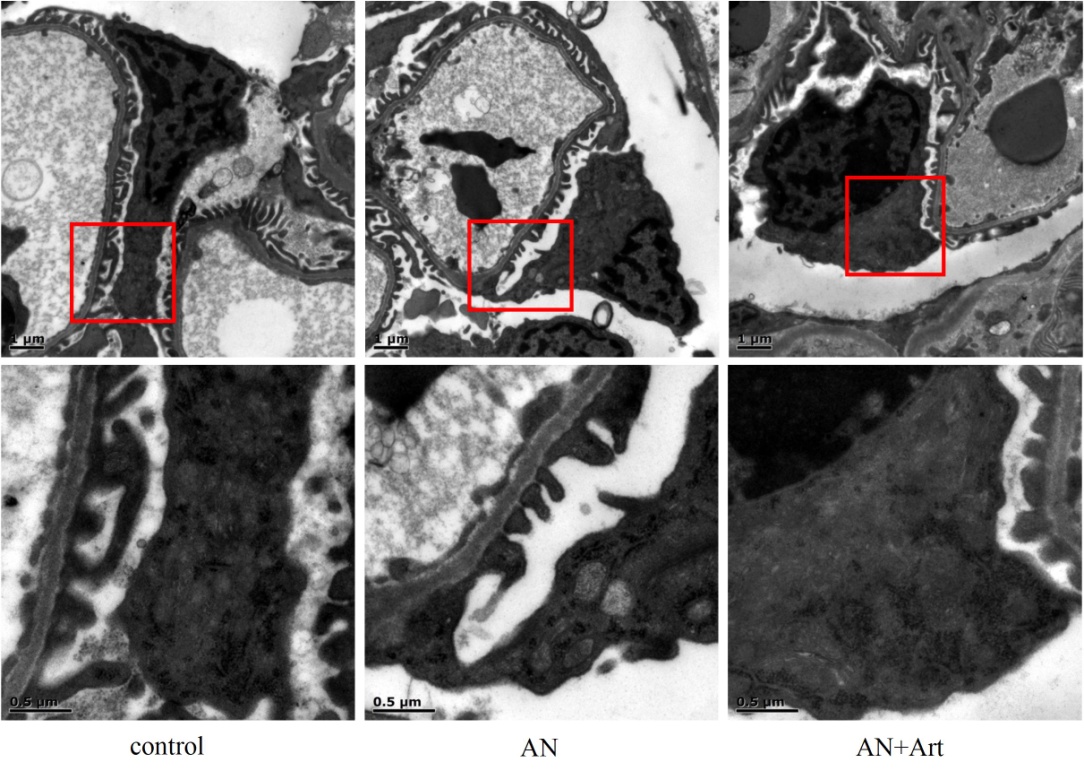


**Figure S4**


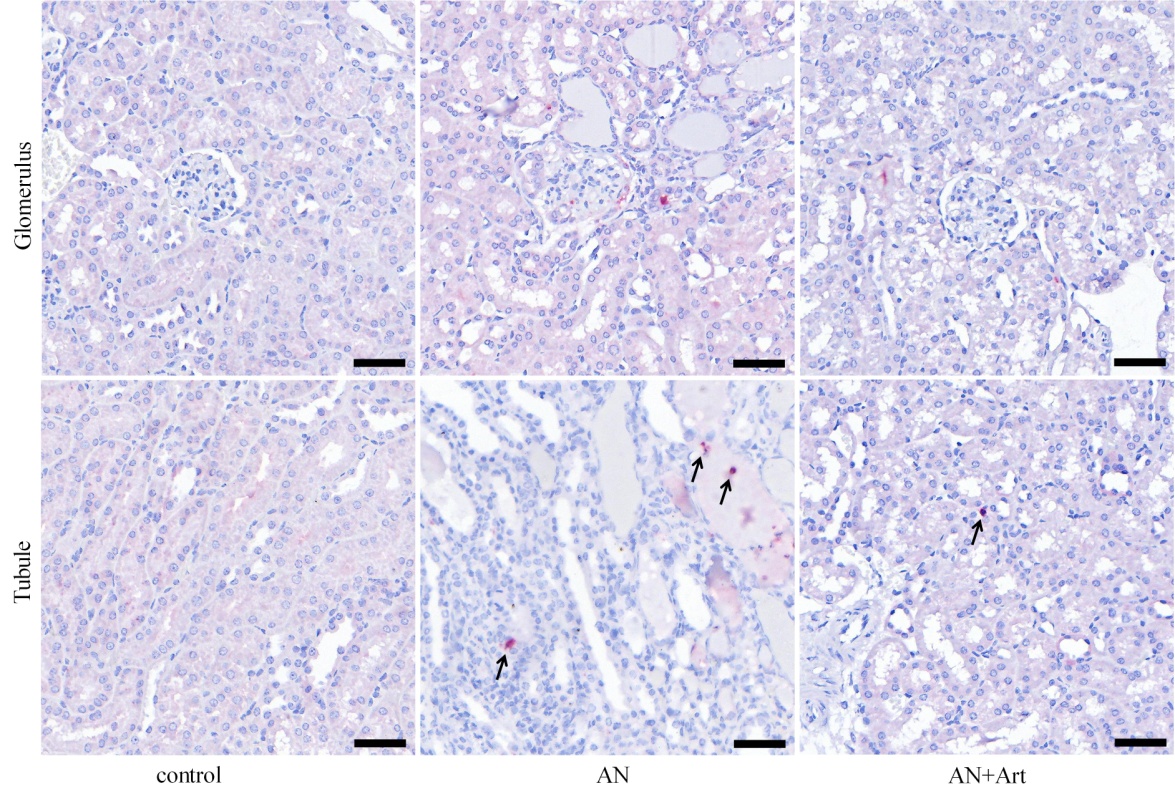


**Figure S5**


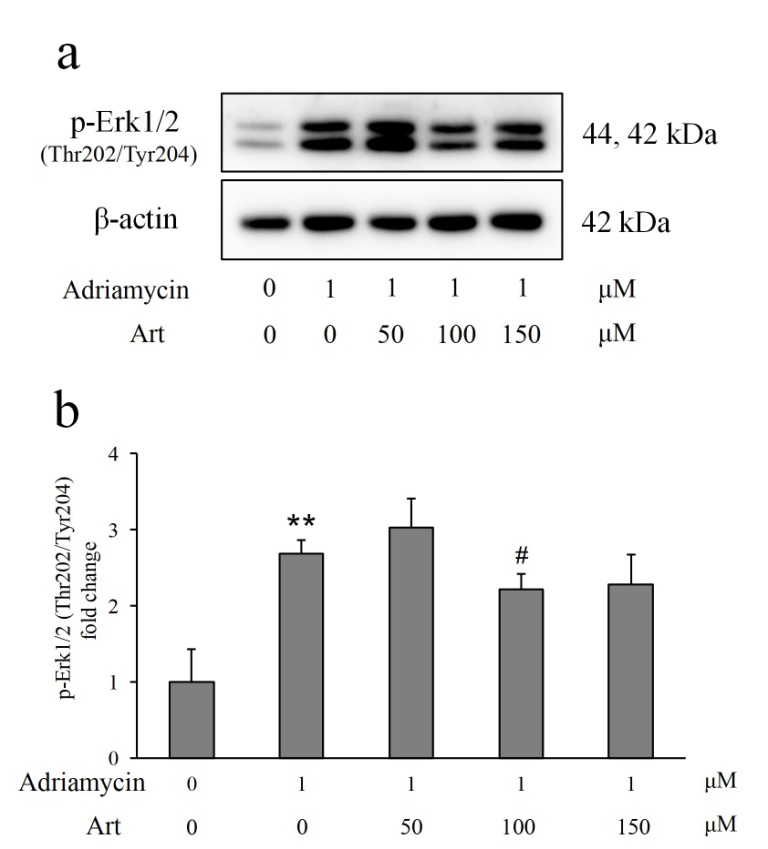


**Figure S6**


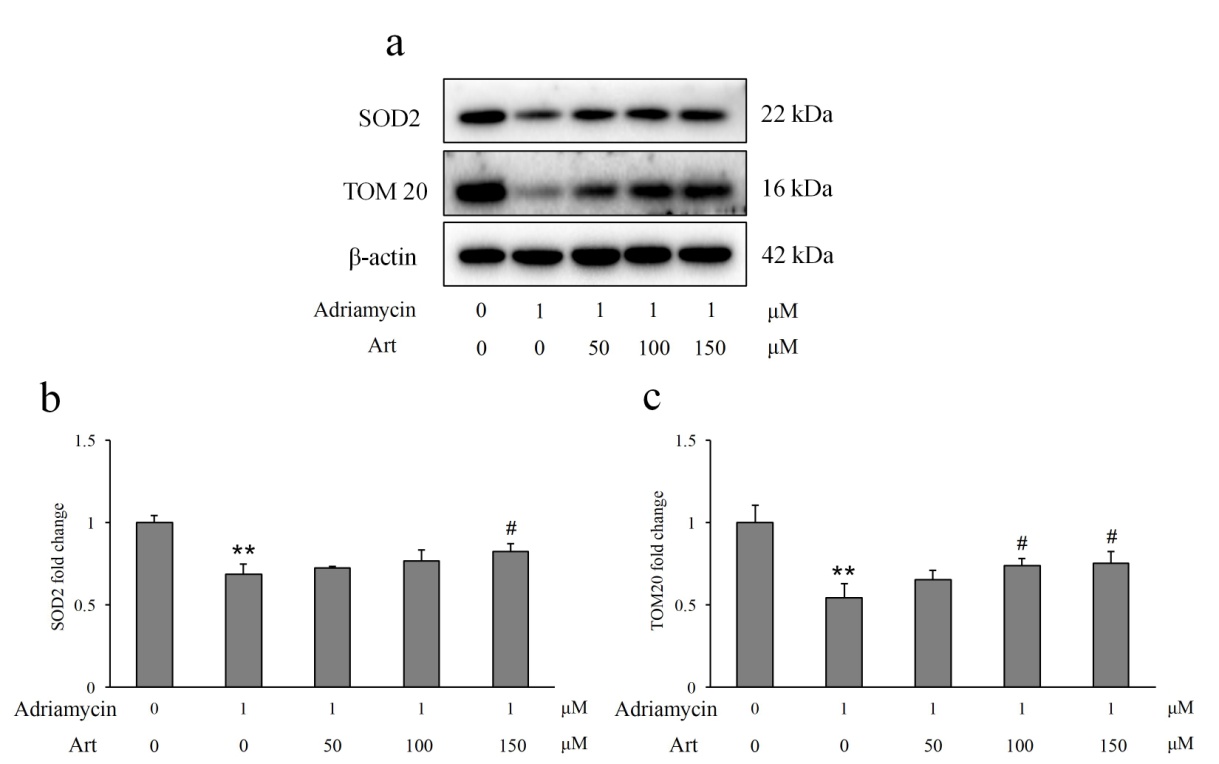


**Figure S7**


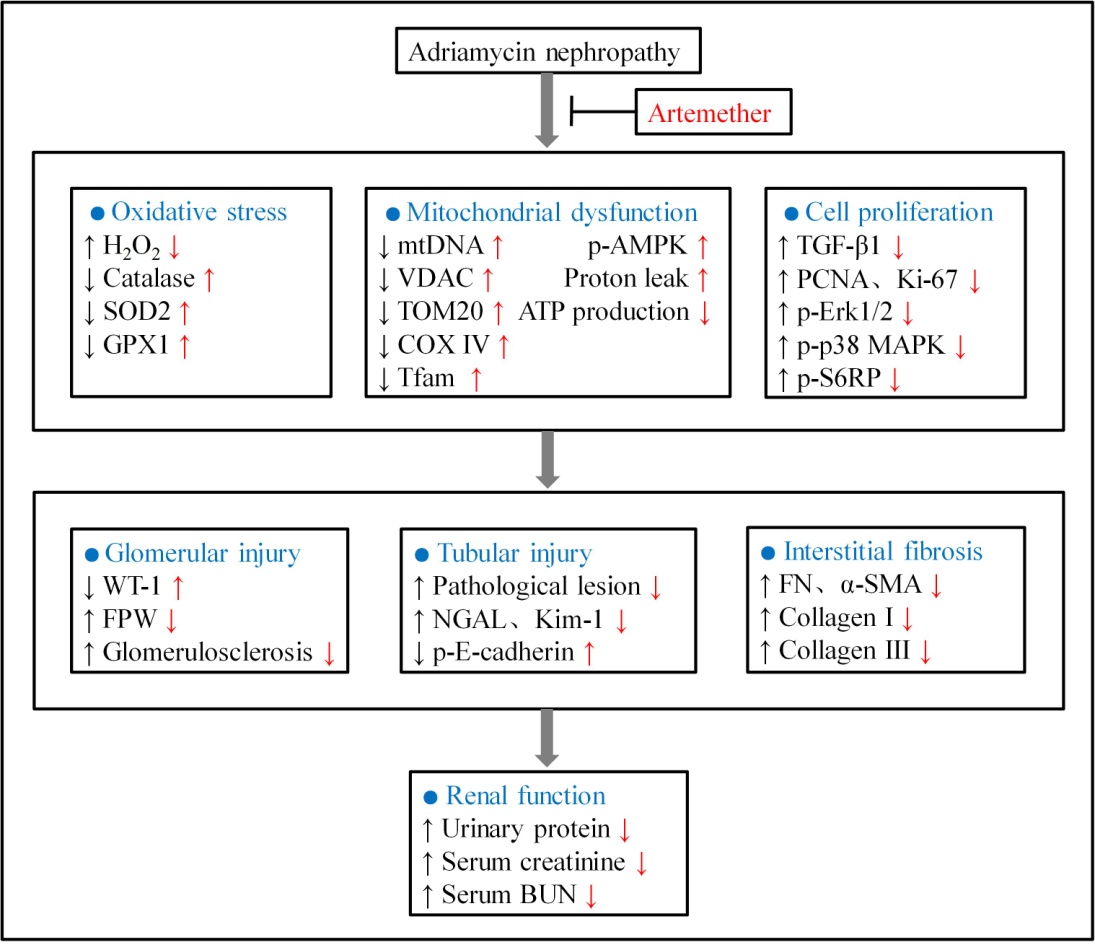

Supplement: Supplementary file 1 — Supplementary Information. [file 41598_2020_80298_MOESM1_ESM.docx]
