# Supplementary material for: Artemether ameliorates kidney injury by restoring redox imbalance and improving mitochondrial function in Adriamycin nephropathy in mice
Source: Sci Rep. 2021 Jan 14;11:1266. doi: 10.1038/s41598-020-80298-x (PMC7809108; doi:10.1038/s41598-020-80298-x)

# Artemether ameliorates kidney injury by restoring redox imbalance and improving mitochondrial function in adriamycin nephropathy mice

**Pengxun Han[1,3], Yuchun Cai[1,3], Yao Wang[1], Wenci Weng[1], Yinghui Chen[1], Menghua Wang[1], Hongyue Zhan[1], Xuewen Yu[2], Taifen Wang[1], Mumin Shao[2,*], Huili Sun[1,*]**

1. Department of Nephrology, Shenzhen Traditional Chinese Medicine Hospital, The Fourth Clinical Medical College of Guangzhou University of Chinese Medicine.

2. Department of Pathology, Shenzhen Traditional Chinese Medicine Hospital, The Fourth Clinical Medical College of Guangzhou University of Chinese Medicine.

3. Pengxun Han and Yuchun Cai contributed equally to this work.

* **correspondence to:** Huili Sun, Department of Nephrology, Shenzhen Traditional Chinese Medicine Hospital, The Fourth Clinical Medical College of Guangzhou University of Chinese Medicine, 1 Fuhua Road, Futian District, Shenzhen 518033, Guangdong, China. Tel: 86-755-83214509, Fax: 86-755-88356033, E-mail: sunhuili2011@126.com

Mumin Shao, Department of Pathology, Shenzhen Traditional Chinese Medicine Hospital, The Fourth Clinical Medical College of Guangzhou University of Chinese Medicine, 1 Fuhua Road, Futian District, Shenzhen 518033, Guangdong, China. Tel: 86-755-23987273, Fax: 86-755-88356033, E-mail: smm026@163.com

## Supplementary Information

### Full-length blots in Figure 4

p-E-cadherin (S838+S840)

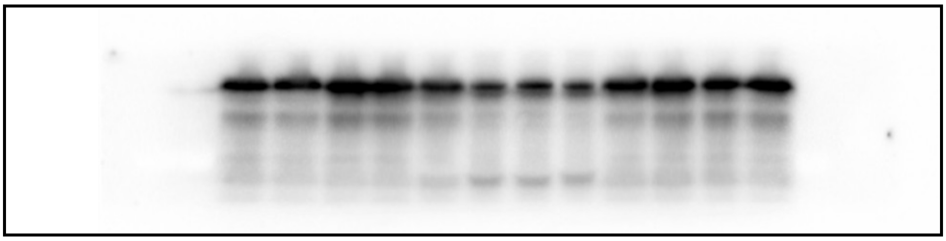

E-cadherin

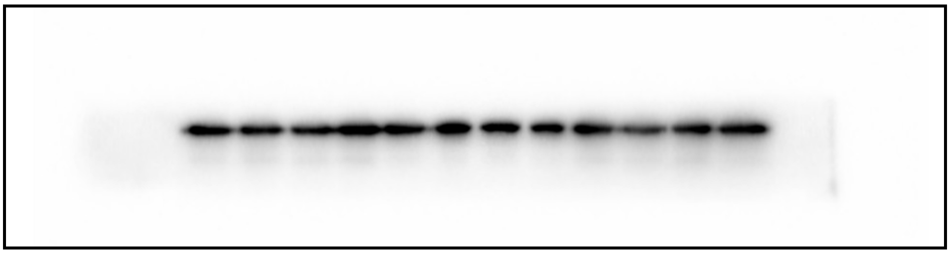

p-Erk1/2 (Thr202/Tyr204)

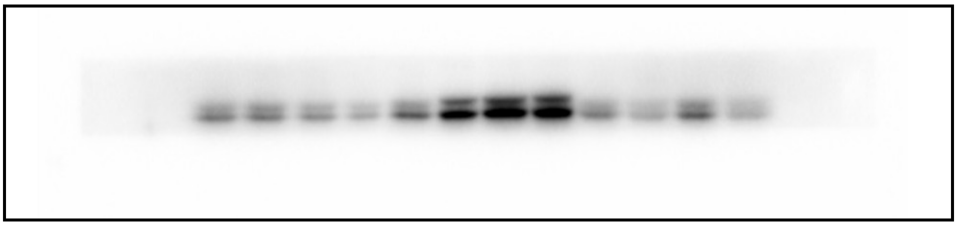

Erk1/2

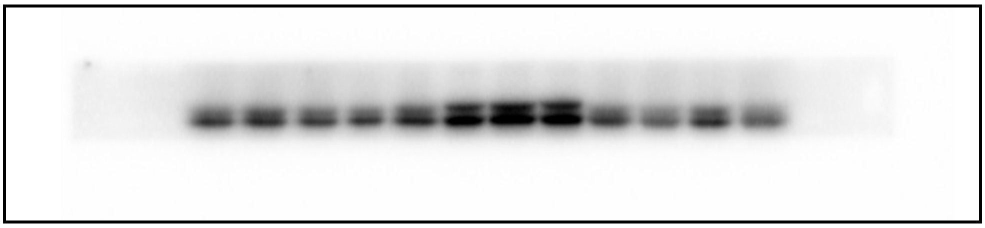

β-actin

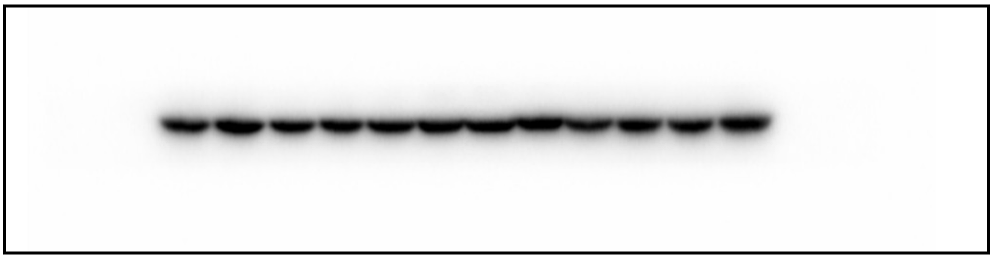

p-p38 MAPK (Thr180/Tyr182)

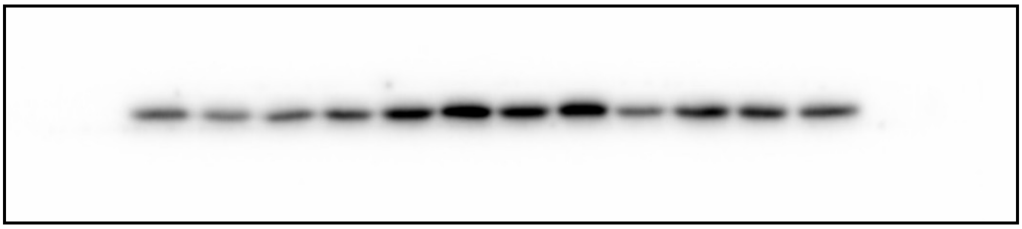

p38 MAPK

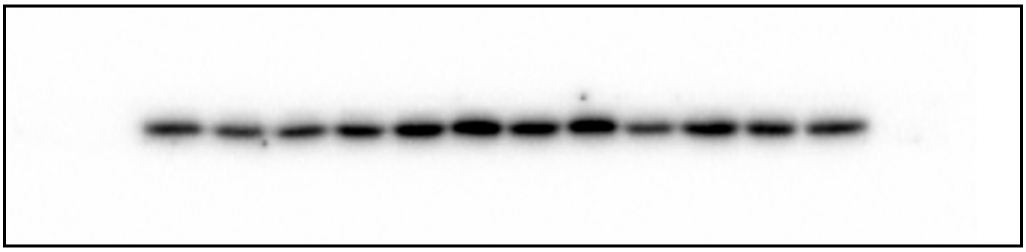

β-actin

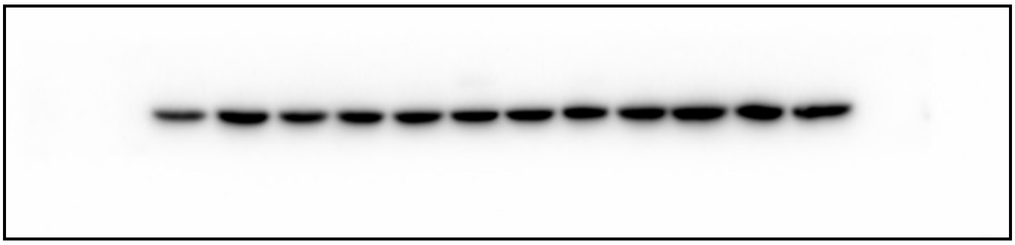

p-S6RP (Ser235/236)

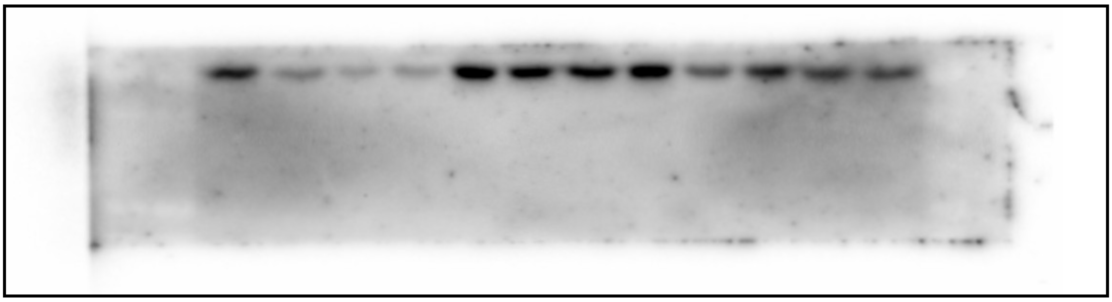

S6RP

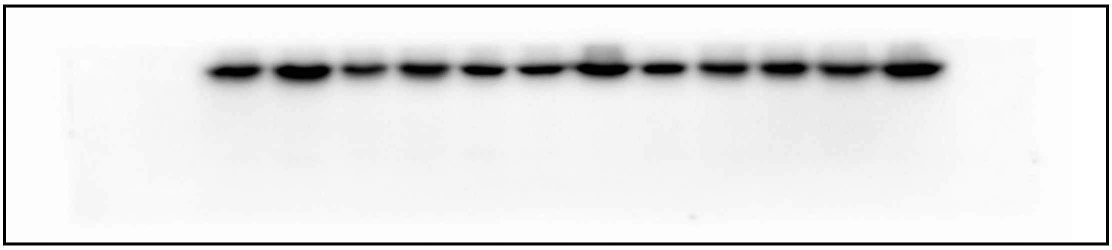

β-actin

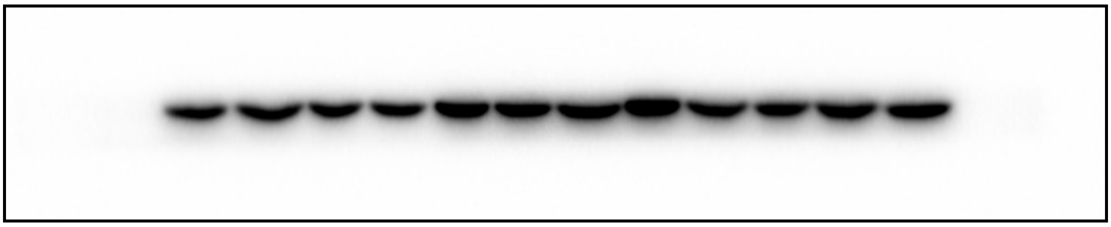

**Full-length blots in Figure 5**

p-Erk1/2 (Thr202/Tyr204)

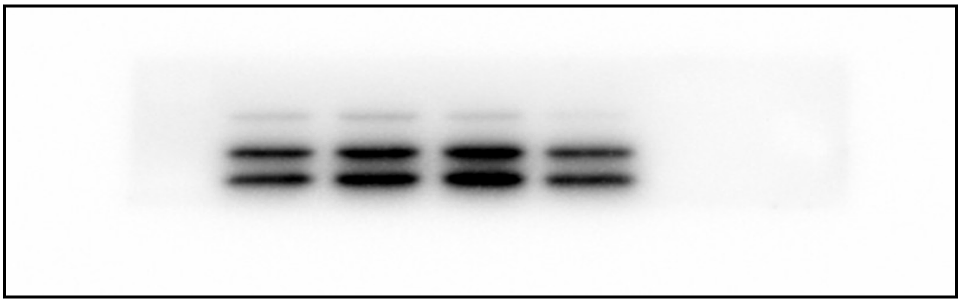

Erk1/2

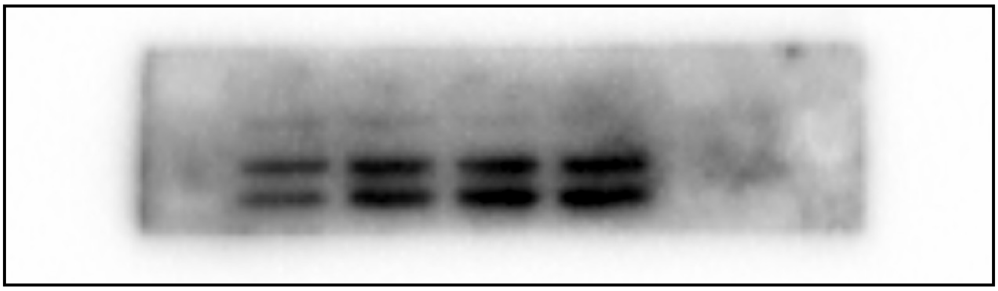

β-actin

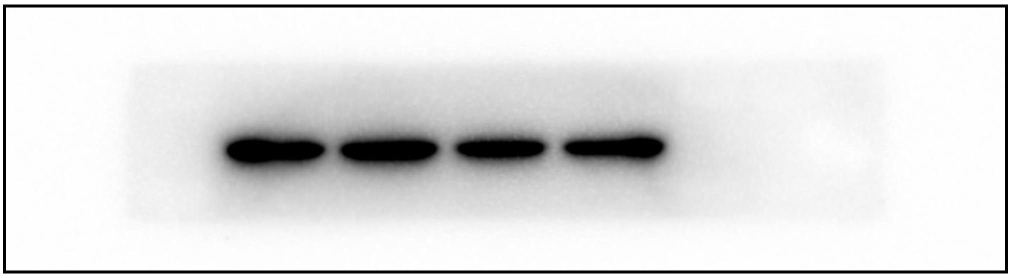

p-S6RP (Ser235/236)

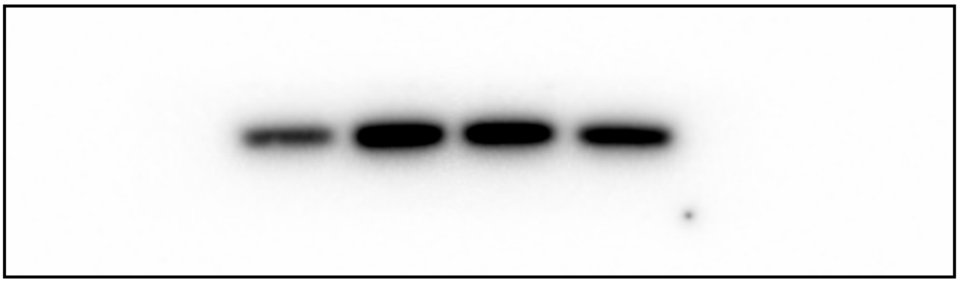

S6RP

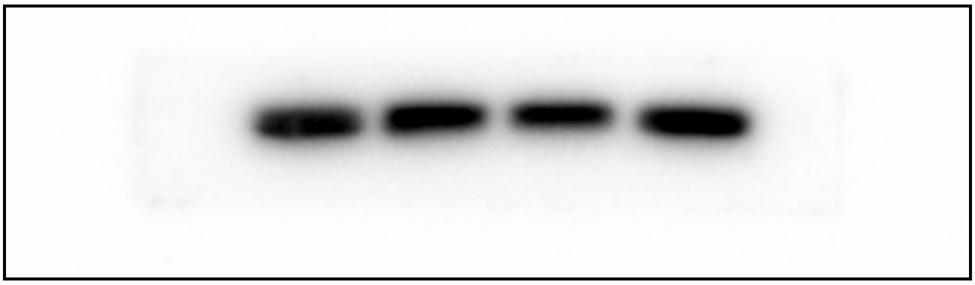

β-actin

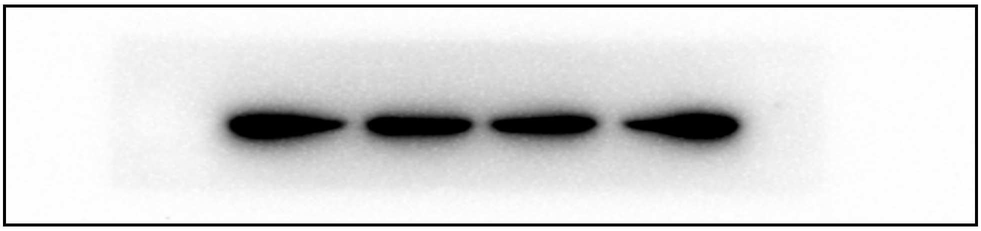

p-p38 MAPK (Thr180/Tyr182)

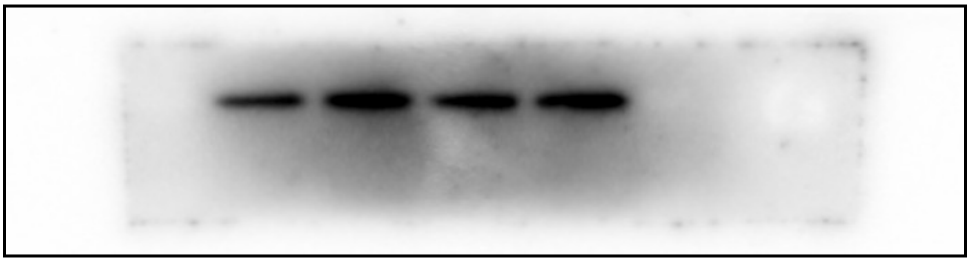

p38 MAPK

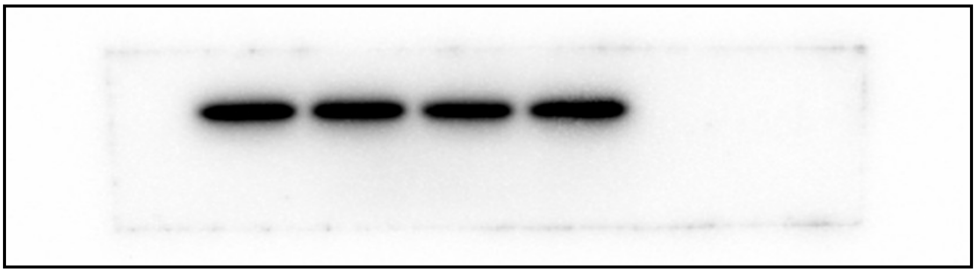

β-actin

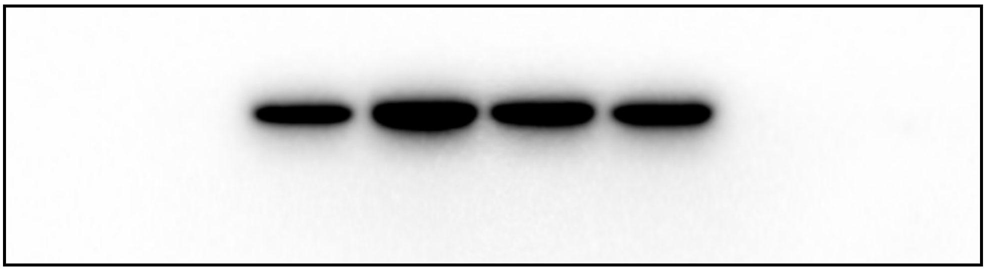

**Full-length blots in Figure 6**

catalase

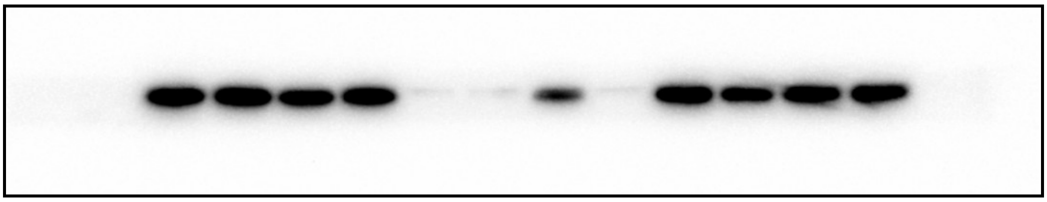

SOD2

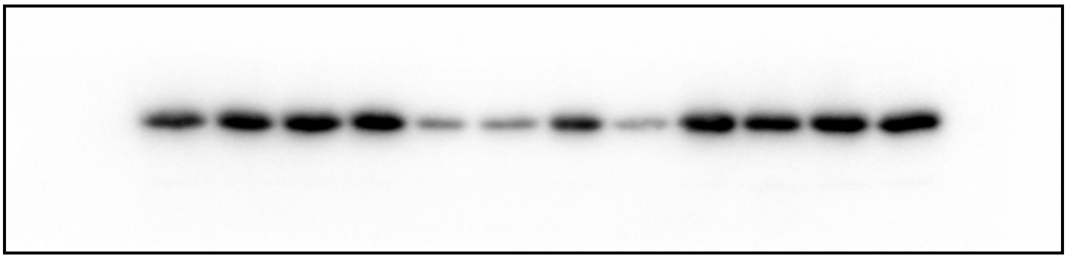

β-actin

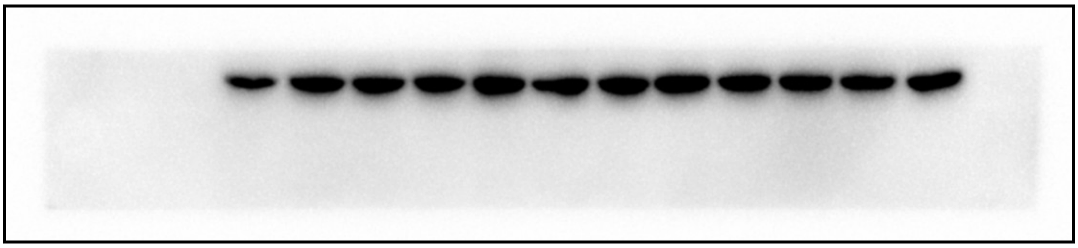

GPX1

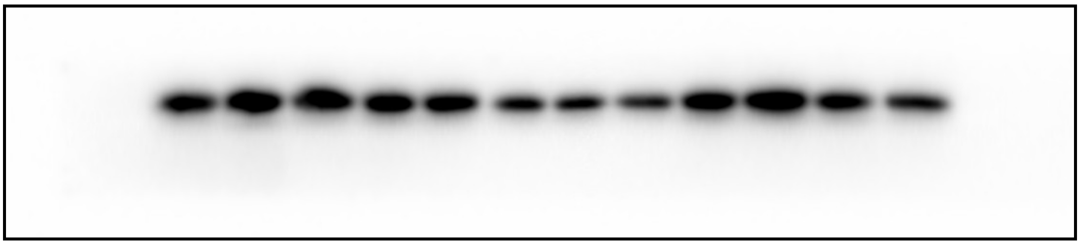

β-actin

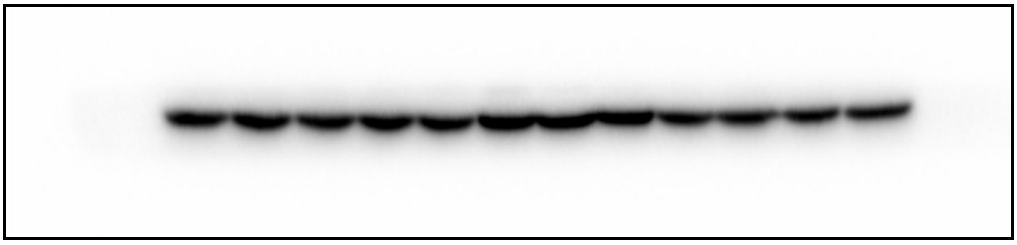

**Full-length blots in Figure 7**

VDAC

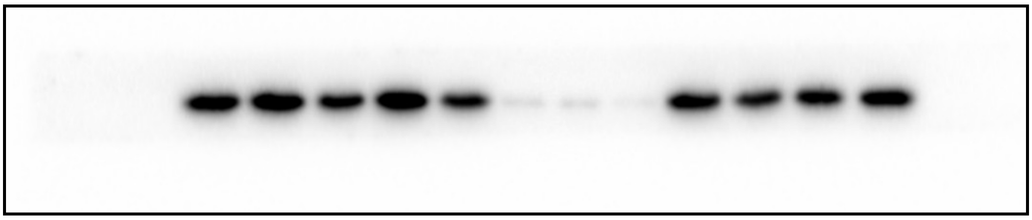

TOM 20

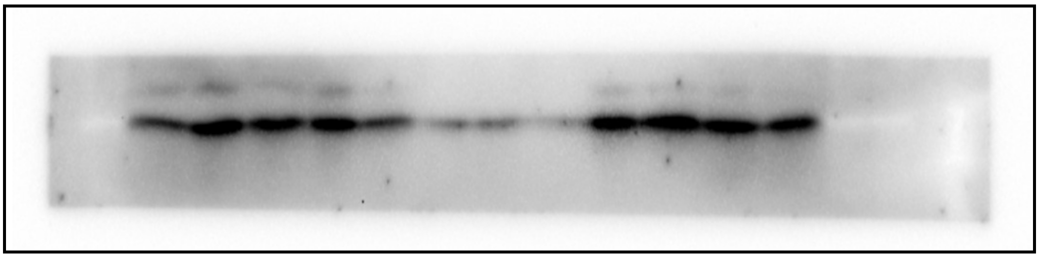

COX IV

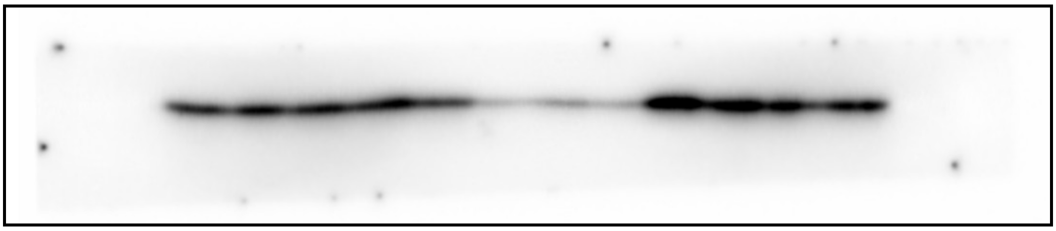

Tfam

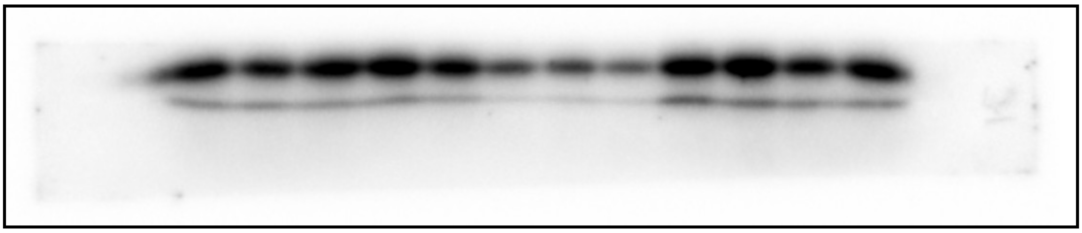

β-actin

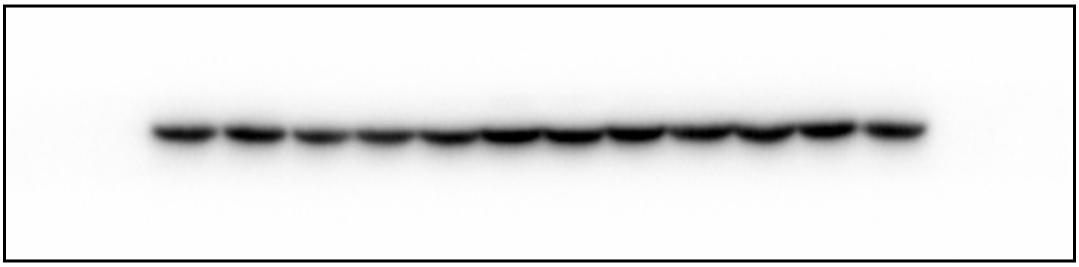

**Full-length blots in Figure 8**

p-AMPK (Thr172)

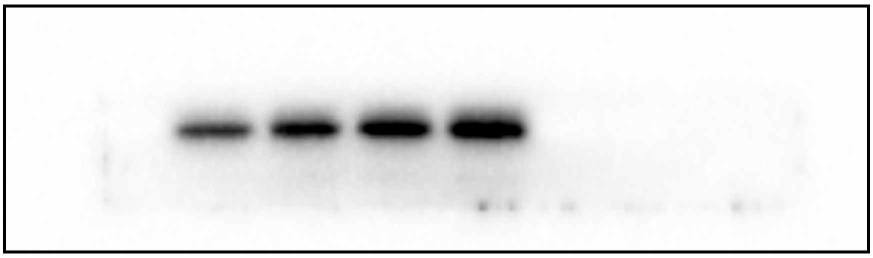

β-actin

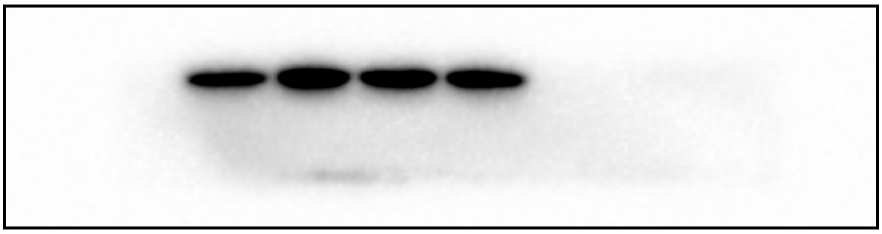

Supplement: Supplementary file 2 — Supplementary Information. [file 41598_2020_80298_MOESM2_ESM.pdf]
